# Ethnoichthyology of freshwater fish in Europe: a review of vanishing traditional fisheries and their cultural significance in changing landscapes from the later medieval period with a focus on northern Europe

**DOI:** 10.1186/s13002-020-00410-3

**Published:** 2020-10-30

**Authors:** Ingvar Svanberg, Alison Locker

**Affiliations:** 1 Institute for Russian and Eurasian Studies, Uppsala University, Box 514, SE-751 20 Uppsala, Sweden; 2 Escaldes-Engordany, Andorra

**Keywords:** Archaeozoology, Ethnozoology, Fishing culture, Fishing practices, Folk biology, Fishponds, Foraging activities, Human-animal relationship, Human nutrition

## Abstract

**Background:**

Fishing is probably one of the oldest economic activities in the history of humankind. Lakes, rivers and streams in Europe are important elements in the European landscape with a rich diversity of fish and other aquatic organisms. Artisanal fisheries have therefore been of great importance for the provision of food, but also animal feed, medicine, fertilizer and other needs. These fishermen had a deep knowledge about the waterscape and its biota. However, ethnoichthyology remains a small topic within contemporary ethnobiology in Europe. Our focus lies within northern Europe in the late medieval to modern period, but encompasses the wider area with some reference to earlier periods where informative.

**Method:**

We have reviewed a large amount of literature mainly on the relationship between man and fish in freshwaters from late medieval times (defined here as the fifteenth century) until the early twenty-first century. The main focus is on freshwater (including anadromous and catadromous) fish in northern Europe, the main area of study for both authors, though examples have been included from elsewhere to indicate the widespread importance of these fisheries. The review includes studies from various fields such as archaeology, ethnography, fish biology, geography, linguistics and osteology to map what has been studied of interest in ethnoichthyology. These data have been analysed and critically reviewed.

**Results:**

There are archaeozoological studies, studies of specialised fishers as well as artisanal fishing among the peasantry, research of folk taxonomies, fishing methods (including the use of poison) and gear, which are all of great interest for ethnoichthyology. There is also research on traditional preserving methods for fish as food and for other purposes. Of interest is the keeping of fish in wells, ponds and aquaria. However, there is still room for more research within many domains of ethnoichthyology.

**Conclusion:**

Humans have always utilized fish and other aquatic resources. Nonetheless, few ethnobiologists working within Europe are so far researching human-fish relationships. This paper demonstrates the range of research available, but also points to future studies. It is important to widen ethnobiological research in Europe to include fish.

## Introduction

This review is an invitation to further the research in the field human-fish relationships, especially the importance of freshwater fish for human culture in a European context. Ethnobiology is a recognized worldwide scientific discipline that can briefly be defined as a diverse research field, which includes many aspects of human relationships with the surrounding biota. Nevertheless, most research in European contemporary ethnobiology has so far concentrated on human-plant relations. However, the biota are much more comprehensive and include many interesting aspects. Therefore, in this review article, we want to highlight the subfield of ethnoichthyology as a research field that deserves more practitioners among European scholars. We want to do so by introducing research from various fields (archaeology, ecology, ethnology, fishery biology, linguistics, zoology) that can serve as a starting point for future ethnoichthyology studies in Europe.

Humans are closely dependent on the waterscape; it provides many ecosystem services, both cultural and provisional [1]. Aquatic resources have always been utilised globally [2, 3] and fishing is probably one of the oldest economic activities in human history [4]. Europe encompasses a great diversity of climates, landscapes and habitats. Lakes, rivers and streams are important elements in the European landscape with a rich diversity in fauna and flora [5]. There are many fish species in European freshwaters; the latest check-list enumerates over 540 species [6] and freshwater fish and fishing have been of immeasurable importance to the rural economy of Europe providing food both locally and for growing urban populations [7–9]. Although commercial fishing targeting a wide range of both freshwater and diadromous fish species now dominates European inland fisheries, small-scale subsistence fisheries still exist in certain areas [10].

Since aquatic resources were readily available for human food, animal feed, fertilizer and countless other purposes, people made the most of them. Fish is a substantial protein and vitamin source in the human diet, and fish management has always been important. Freshwater fish have also had significant cultural value both in fishers’ folklore and as part of traditional cuisine marking certain dates in the calendar. To study the interactions between fish and people, it is vital to understand the history of these interfaces.

Europe, like the rest of the world, is rapidly changing environmentally and culturally and much local knowledge is already lost. This has implications for modern fisheries as local knowledge from personal investment in fisheries makes a vital contribution to current and future fisheries policies as fish stocks collapse [11]. Through archaeological, historical and ethnographic sources, it is possible to study otherwise little known practices of the past that also impinge on the present.

Research dealing with the dynamic interrelationship between human beings and other species in the surrounding biota is the subject for ethnobiologists [12, 13]. Faunal remains recovered from archaeological excavations from prehistoric times onwards are studied by archaeozoologists/zooarchaeologists [9]. No matter what we call ourselves, our common goal is to study human co-evolution, our relationships and mutual influence with other species through human history [14]. Although, as Steve Wolverton stresses, our research field comes from various disciplines, ethnobiology (here, we include archaeozoology) is not a subfield of any traditional disciplines, but bridges humanities, social science and science [15]. In brief, our research field is found at the intersection of what we routinely call ‘culture’ and ‘nature’.

Our choice of data has, of necessity, been selective and is influenced by the authors’ areas of study. However, specific examples from areas elsewhere in Europe demonstrating a long history and tradition of freshwater exploitation have also been included to emphasise the importance of this resource through time and space.

## The scope of ethnoichthyology

### Background

Ethnoichthyology is defined as a subfield within the discipline ethnobiology [16, 17]. The American anthropologist Warren T. Morrill first coined the term in 1967. He researched the naming, knowledge and use of fish among Creole-speaking Chacha islanders in Saint Thomas, U.S. Virgin Islands [18]. The term ethnoichthyology was immediately accepted by his younger colleague, Eugene N. Anderson, who researched fishing culture among the boat people in Castle Peak Bay in Hong Kong’s New Territories. Anderson’s dissertation is a meticulously detailed analysis of how Cantonese-speaking boaters classify known species of fish [19].

These two ground-breaking studies have contributed to our theoretical understanding of the complexities underlying popular classification systems of aquatic resources. They have also inspired a few other scholars to follow in their footsteps. Ethnoichthyological fieldwork has been conducted in Africa, Asia and Oceania, e.g. [20–22]. Reinman’s work on fishing in Oceana was an early archaeological approach, looking at the material evidence with reference to earlier anthropological studies to ‘enable the archaeologist to see fishing as a functioning part of the economic picture’ [23]. Hilary Stewart’s study on methods of catching, storing and cooking fish and their place in local mythology through her observations and conversations with First Nation peoples on the northwest coast of North America, first published in 1977, remains a classic reference work [24]. An important precursor, which dealt with the importance of fish and fishing among Native Americans, was published by Erhard Rostlund in 1952 [25]. Although the field is not as popular as for instance ethnobotany, in Brazil, ethnoichthyology seems to be a flourishing research field and a number of colleagues have published studies that illustrate different aspects of human relationships with fish, e.g. [26–28].

Ethnoichthyology focuses especially on local knowledge, linguistic expressions, nutritious importance, folk practices, material evidence and cognitive perceptions of fish and the environmental consequences of these interactions. There is a long history of fish introduced to new waters by humans, deliberately or accidentally, while others disappear or become extinct. Sometimes, the introduction of one species leads to the extirpation of another.

### European interest

Very few scholars doing research in European waters have so far characterized themselves as ethnoichthyologists or ethnobiologists [29–33]. However, long before ethnoichthyology (and ethnobiology) were established as disciplines in Europe, anthrogeographers**,** ethnologists, fishery biologists, folklorists, historians, linguists and osteologists carried out research on the importance of fish and fishing. Ethnologists documented fishing techniques, fishing gear and fishing communities [34–37]. They focused on the catching methods, fishing gear and customs of artisanal fishing. In choosing and developing fishing gear, people have shown great ingenuity based on their knowledge of fish behaviour. The Polish ethnologist Kazimierz Moszyński, who was interested in local ways of categorizing fish species, as well as fishing gear and techniques, concluded that fishing practices among Slavic peoples had a long and shared lineage as many fish names sound similar in all Slavic languages [38]. The Bosnian archaeologist and ethnologist Vejsil Ćurčić’s (1868–1959) study of inland fishing pre-World War 1 is another example, which could serve as a an exemplary model for contemporary ethnoichthyological research, since it deals not only with fishing gear and techniques but also names, ecological and other aspects of the human-fish relationship [36].

Many fishing methods and gear still in use are of ancient origin [39] as archaeological remains of wooden fish traps, nets and weights preserved in waterlogged conditions and old illustrations and descriptive texts confirm. Surviving old fishing techniques have continued to fascinate ethnographers when they have encountered them in the field [40–44]. Among a younger generation of researchers, the Finnish ethnologist Nils Storå chose an ecological approach to understand the importance of fish for Åland islanders [45].

Fishery biologists have documented important details of traditional fisheries and their prosecution in lakes and rivers. They also gathered local ichthyonyms as well as local ecological knowledge among fishermen. Several of these studies are rich in interesting details [46–50]. Some fish monographs dealing with historical aspects include interesting data on traditional fishing of certain species, such as the sturgeon in the River Rhine and the huchen, also known as the Danube salmon, in Polish rivers [51, 52]. In Sweden, as early as the late nineteenth century, fishery biologists gathered data by using questionnaires [53].

In addition, folk beliefs have been studied [54] and linguistic elements such as folk ichthyonyms [55–58]. Toponyms are another aspect reflecting human-fish relationships and the landscape/waterscape of fishing culture [59–61]. Also other linguistic aspects, such as the local vocabulary of fishing, are all of interest for ethnoichthyologists [62, 63].

Within cultural and social anthropology, there was widespread interest in marine fisher cultures in the 1970s and 1980s. Many of these studies focused on human relationships with fish; some explicitly included ethnoichthyological aspects. For an extensive review up to the early 1980s, see James M. Acheson [64] and for a more recent review, published in 2020, see Fiona McCormack and Jacinta Forde [65].

## Purpose

**Fig. 1:**
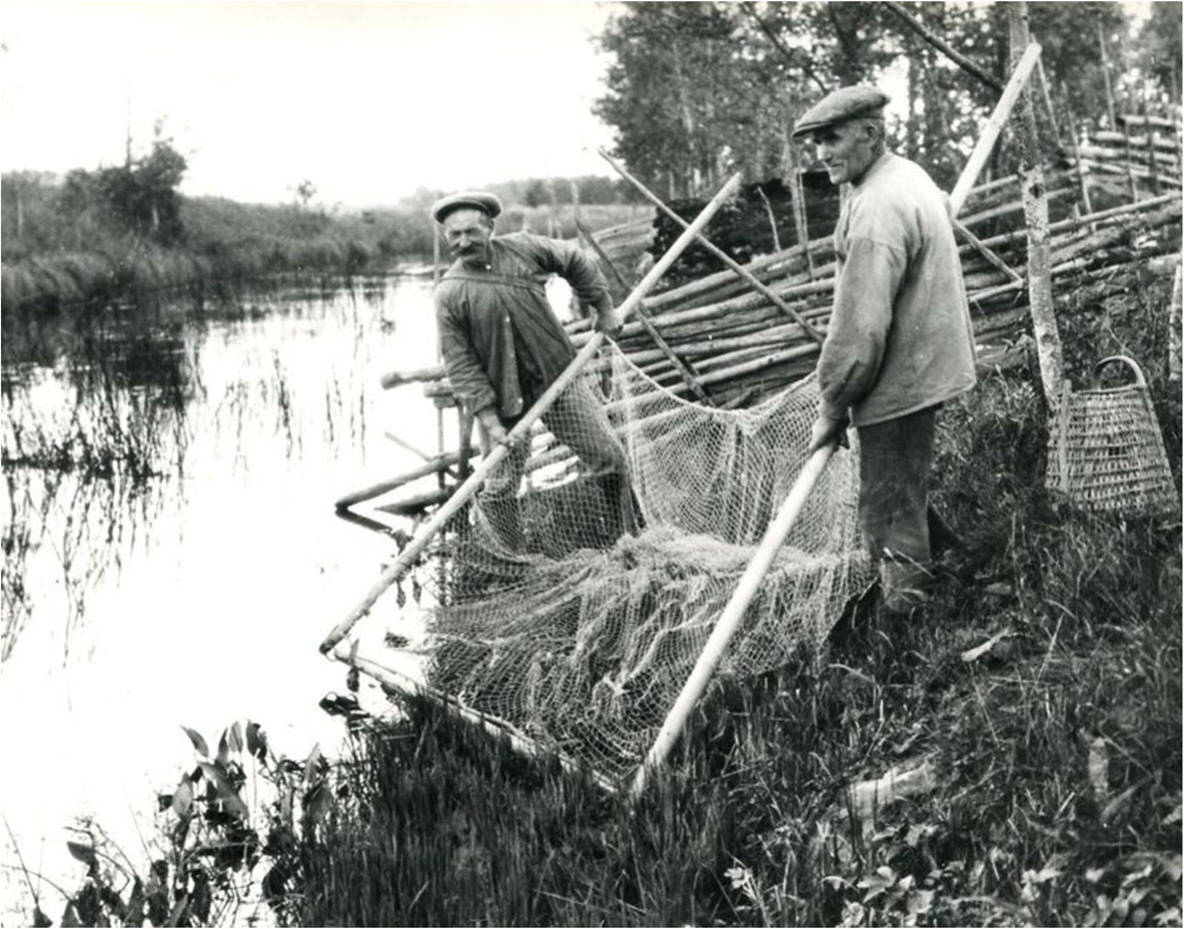
Fishing gear used by local farmers in a stream outside Uppsala in the early twentieth century. It was an illegal fishing method when the photo was taken by Ivar Arwidsson. However, the local villagers still used it at least once a year (Courtesy: Nordic Museum, Stockholm)

For the last few decades, there have been an increasing number of ethnobiologists in Europe primarily dealing with the relationships between plants and people, i.e. ethnobotany [13, 66]. In this paper, we want to emphasise the importance of studying human interaction with other organisms specifically the importance of fish and fishing in the recent past and present of peoples in Europe, which should not be underestimated (Fig. 1).

Harvesting of fish has always influenced people’s choice of residence, livelihood, communications, economy, cultural expressions, language use, material culture and social conditions. The landscape as well as the waterscape is still very much influenced by human-fish relationships. The fauna has also been and continues to be affected by these complex activities in many areas within Europe and should therefore be of interest. Europe, defined as stretching from the Arctic Ocean to the north, the Atlantic Ocean to the west and the Mediterranean, Black and Caspian Seas to the south, and the Ural River in the east, includes rich marine ecosystems characterised by waters with a high salt content and their own ethnoichthyological history. However, in this article, we primarily deal with fish that are generally perceived as freshwater fish, but also include anadromous and catadromous fish, as well as some coastal species found in brackish waters such as estuaries and brackish seas (Table 1).

**Table 1:** Fish mentioned in the text

Family Petromyzontidae	
River lamprey	*Lampetra fluviatilis* (L., 1758)
Family Acipenseridae	
Sturgeon (European)	*Acipenser sturio* (L., 1758)
Family Anguillidae	
Eel (European)	*Anguilla anguilla* (L., 1758)
Family Clupeidae	
Herring (Atlantic)	*Clupea harengus* L., 1758
Baltic herring	*Clupea harengus* L., 1758
Sprat (European)	*Sprattus sprattus* (L. 1758)
Shad	*Alosa* sp.
Family Salmonidae	
European whitefish, powan	*Coregonus lavaretus* (L., 1758)
Vendace	*Coregonus albula* (L., 1758)
Atlantic salmon	*Salmo salar* L., 1758
Brown trout (and sea trout)	*Salmon trutta* L., 1758
Rainbow trout	*Oncorhynchus mykiss* (Walbaum, 1792)
King salmon	*Oncoryhnchus tshawytscha* (Walbaum, 1792)
Red salmon (sockeye)	*Oncoryhnchus nerka* (Walbaum, 1792)
Chum salmon	*Oncoryhnchus keta* (Walbaum, 1792)
Silver salmon (coho)	*Oncoryhnchus kisutch* (Walbaum, 1792)
Pink salmon	*Oncoryhnchus gorbuscha* (Walbaum, 1792)
Grayling	*Thymallus thymallus* (L., 1758)
Arctic char	*Salvelinus alpinus* (L., 1758)
Huchen (Danube salmon)	*Hucho hucho* (L., 1758)
Family Osmeridae	
Smelt (European)	*Osmerus eperlanus* (L., 1758)
Family Esoxidae	
Pike (Northern)	*Esox lucius* L., 1758
Family Umbridae	
European mudminnow	*Umbra krameri* (Walbaum, 1792)
Family Cyprinidae	
Common carp	*Cyprinus carpio* L., 1758
Crucian carp	*Carassius carassius* (L., 1758)
Goldfish	*Carassius auratus* (L., 1758)
Tench	*Tinca tinca* (L., 1758)
Bream	*Abramis brama* (L. 1758)
Vimba bream	*Vimba vimba* (L., 1758)
Bleak	*Alburnus alburnus* (L. 1758)
Barbel	*Barbus barbus* (L. 1758)
Andalusian barbel	*Luciobarbus sclateri* (Günther, 1868)
Gudgeon	*Gobio gobio* (L. 1758)
Nase	*Chondrostoma nasus* (L. 1758)
Bitterling	*Rhodeus amarus* (Bloch, 1782)
Asp	*Leuciscus aspius* (L., 1758)
Dace	*Leuciscus leuciscus* (L. 1758)
Ide	*Leuciscus idus* (L. 1758)
Chub	*Squalius cephalus* (L. 1758)
Minnow	*Phoxinus phoxinus* (L. 1758)
Roach	*Rutilus rutilus* (L., 1758)
Sunbleak	*Leucaspius delineatus* (Heckel 1843)
Topmouth gudgeon	*Pseudorasbora parva* (Temminck & Schlegel, 1846)
Family Copidae	
Weatherfish	*Misgurnus fossilis* (L., 1758)
Stone loach	*Barbatula barbatula* (L., 1758)
Family Siluridae	
Wels	*Silurus glanis* L., 1758
Family Gadidae	
Cod (Atlantic)	*Gadus morhua* L., 1758
Burbot	*Lota lota* (L., 1758)
Ling	*Molva molva* (L., 1758)
Family Gasterosteidae	
Three-spined stickleback	*Gasterosteus aculeatus* (L., 1758)
Family Percidae	
Perch (European)	*Perca fluviatilis* L., 1758
Ruffe	*Gymnocephalus cernua* (L. 1758)
Pike-perch (Zander)	*Sander lucioperca* (L., 1758)
Family Cichlidae	
Nile Tilapia	*Oreochromis niloticus* (L., 1758)
Family Pleuronectidae	
Flounder	*Platichthys flesus* (L., 1758)
Family Osphronemidae	
Paradise fish	*Macropodus opercularis* (L., 1758)
Siamese fighting fish	*Betta splendens* Regan 1910
Family Poeciliidae	
Mosquito fish	*Gambusia affinis* (Baird & Girard, 1853)
Family Scombridae	
Blue fin tuna	*Thunnus thynnus* (L. 1758)

Source: FishBase.org

The purpose of this article is to review and discuss the importance of further research on the interrelations between humans and freshwater fish in the European context. Bērziŋš made a study of prehistoric fishing in Eastern Baltic lakes as a year round activity (detectable through fishing gear and vertebral annuli) which could have stimulated permanent settlement for hunter-gatherers [67]. A recent paper has taken a broader global view focussing on global and marine fisheries as they make up the bulk of fish catches [68]. Here, we concentrate on the late medieval period to the early twenty-first century; it is by necessity selective as the geographical and even this limited temporal scope in detail would fill a whole volume.

## Results

### Review and discussion of evidence

#### What can fishbone tell us?

**Fig. 2:**
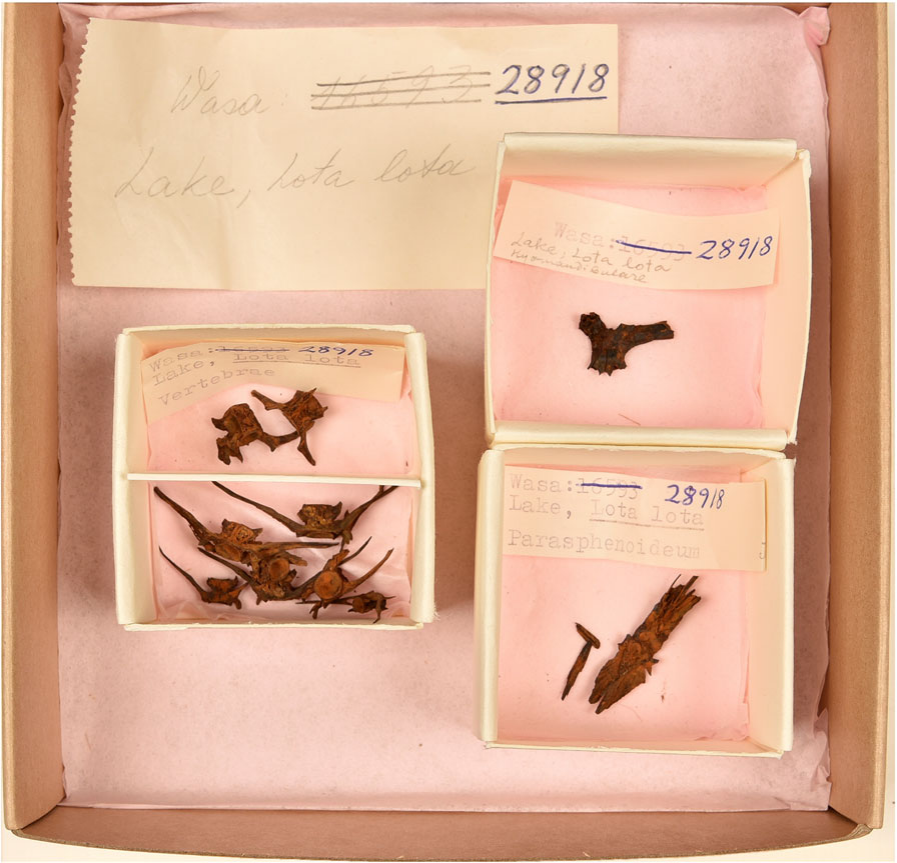
Fish bones of burbot, *Lota lota* (L.), from the Swedish warship Vasa that sank during its maiden voyage in 10 August 1628. Among the provisions for the crew on-board were dried and salted fish, whose bones have been identified to species thus giving as a good insight in popular food fish within the navy in the seventeenth century. Burbot is still eaten in early spring in Sweden (Photo: Kristin Ytterborg, The Vasa Museum, Stockholm)

The sieving of selected archaeological deposits has become increasingly standard practice since the 1970s, designed to recover plant material, insect remains, small bones and microlith debitage. Standard methods include wet sieving sampled deposits through a stack of increasingly smaller mesh sizes to at least 1 mm. The sieve and flotation residue are sorted under magnification, ensuring the recovery of the smallest finds including fish bones. These are often fragmentary and non-specific such as ribs and fin rays while others, including vertebrae and skull bones, may be identifiable to family or species level aided by comparison with reference material. From these data and in conjunction with documentary sources and comparison with other fish assemblages, we can suggest the contribution of different fishes in the diet, their size (through metrical comparison), source and significance at that particular site, region and for the period. In the later medieval period, marine fish were deliverable to many parts of Europe as reflected in the bone data and freshwater fish decreased in importance as part of the fish supply. England with easily accessible coastlines had met the demand for fish with marine supplies from as early as the late tenth century [69]. However freshwater fish continued to play a significant role by status rather than volume including elite cultural contexts, such as private ponds on country estates (Fig. 2).

Fish bones can add to our knowledge about water conditions as well as culinary preferences. Evidence from the growing discipline of ichthyoarchaeology is increasingly used by fish biologists as the bone evidence predates written fisheries records. This provides a longer backstory to fish exploitation and the evidence is used in determining modern fisheries policies. The finds of pollution-sensitive fish such as grayling in archaeological contexts are an aid to mapping the history of deterioration in local river systems. Species identified from sites outside their native range such as common carp and crucian carp complement documentary data to determine the earliest dates of introduction [9].

#### Fisher-forager cultures and peasant fishermen

In late medieval times (our starting point), freshwater fishing had, for some centuries, been subject to legislation to protect young stock and migrating spawning fish from over exploitation, though this resource has long been utilised, since early prehistory. Archaeologists have identified fishing cultures in prehistoric societies in Western Europe, for example, among Magdalenian hunter-gatherers in France and Northern Iberia both through bones and art [70], while the Mesolithic period in Scandinavia was also rich in fishing communities [71]. These fisheries continued to be exploited through the millennia, not least in northern Europe. Artisanal fishing was of great importance in the historic period for the Sami [72], whose specialised fisher-hunter-forager cultures existed until recently along northern rivers and larger lakes in Sweden and in Finland. The Fisher-Sami fished the lakes of Tjäurajaure and Tjieggelvas in Swedish Lapland [73] and the Inari Sami were predominately fishermen in Lake Inari in northern Finland until recently. The Skolt Sami in the borderland between Russia and Finland were mainly fishers before the Second World War. Like other fishing Sami, they migrated along rivers to their camp sites following the fishing season and although this practice has declined, many Skolt Sami are still fishermen [74]. Although we have rich ethnographical and linguistic data from the Sami, ethnoichthyological aspects need further research [75].

If we include also the Russian Far East, there are further ethnic groups subsisting mainly on fishing. A culture relying on the salmon harvest was the Itel'men of the Kamchatka Peninsula, who primarily fished rivers. Despite later integration after Russian colonization at the beginning of the eighteenth century, they continued to practice their traditional intensive fishing. Semi-sedentary in riverside villages, they fished March to October exploiting several runs of different *Oncorhynchus* salmon species: king, red, chum, silver and pink*.* The whole community was involved in catching and processing this essential food store for the winter months using weirs, nettle fibre nets and traps. They made *yukola* (dried fish), mainly from chum salmon whose run took place before the rainy weather, which is detrimental to drying, also fermented fish seasoned in the ground. *Yukola* was for everyday consumption, the fermented fish a delicacy for special occasions. Any waste including bones was fed to their sledge dogs, who were largely fed on pink salmon and enjoyed a ‘soup’ of fermented fish on days they were not working. These communities were dependent on a bountiful fish run (which was subject to natural fluctuations) and good drying conditions for the *yukola*, which was susceptible to rain and fly infestation [76].

**Fig. 3:**
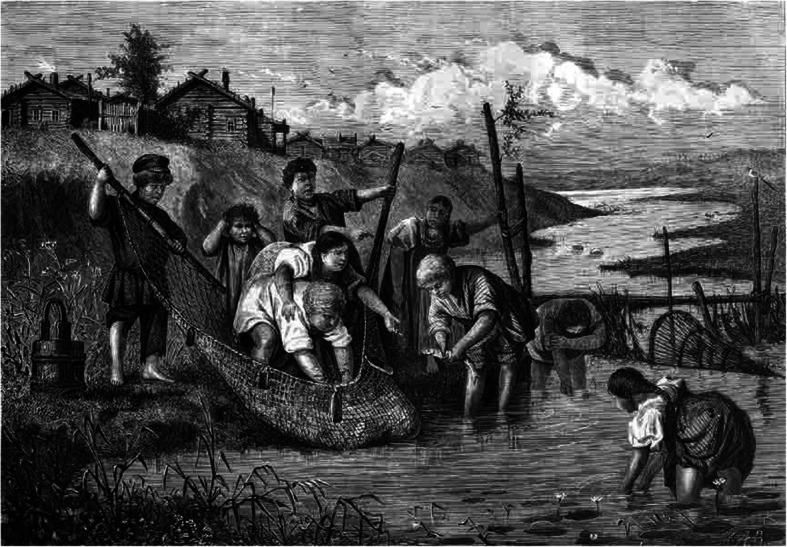
Russian children fishers. Engraving by A.I. Zubchaninov, drawing by A.P. Koverznev, 1875 (From Vsemirnaya Illyustratsia 1875)

Fishing has always been of great importance for the peasantry along the coastlines and those living along rivers and lakes. Anadromous species, such as Atlantic salmon, sea trout, shad and smelt, have had special economic importance providing a seasonal glut for fishermen during their migration [7, 40, 77], while non-migratory fish gather during spawning periods. Important fisheries took place along the rivers Torne and Donau, the Curonian Lagoon, the Masurian Lake District, Lakes Ladoga, Vygozero, Prespa, Ohrid, Skadar, Vänern, Vättern, Mälaren, Saimen, Oulujärvi, Onega, Peipus, Constance, Windermere, Loch Lomond and Lago di Garda, to name but a few. Although we have historical and ethnographical information from some of these areas, there is certainly room for further fieldwork stressing the ethnoichthyological aspects (Fig. 3).

In some areas of Europe, there are peasant fishermen, living in communities that tilled the land but focused more on fishing and gathering. These occupations were seasonal and individually insufficient, but together provided a living. This way of life has a long history; a medieval example is that of the farmer fishers of the south Devon coast in England, who had secondary seasonal settlements for fishing along the coastline and in estuaries. These ‘cellar settlements’ were used for storing fishing equipment, smoking fish and replaced by permanent fishing villages from the end of the fifteenth century [78]. The fisher farmer lifestyle was found along many parts of the British coastline, practiced most recently in remote islands such as Orkney and Shetland off the Scottish northern coast [79] and the now depopulated St Kilda west of the Outer Hebrides [80]. In these locations, survival through traditional crofting relied on a combination of practices including agriculture, livestock, weaving, sea birds and fish [81]. Fisher farmer communities were also found on islands in the archipelagos of the Baltic Sea, an example is Runnö in Kalmar Strait in Sweden, which has been studied by John Granlund. Peasant fishermen on the island combined agriculture, livestock and fishing to form a sustainable integrated lifestyle. However, during the nineteenth century, fishing increased in importance. They fished a variety of species: eel, Baltic herring, pike, salmon, flounder, cod and ide for their own consumption. With the increasing importance of cash assets in the early twentieth century, they focused on eel fishing, which was in more demand and became specialised fishermen [82].

**Fig. 4:**
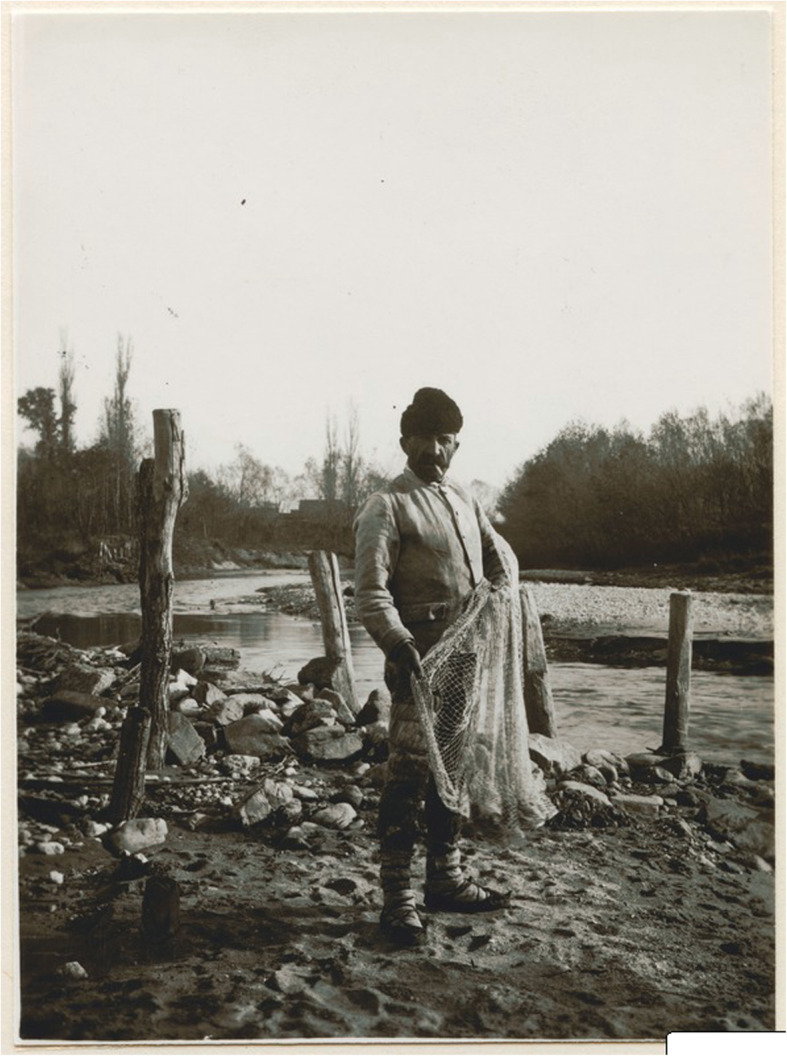
Fisherman with a cast net in the village Neppendorf (Turnișor) near Hermannstadt (Sibiu), Siebenbürgen, Roumania, in 1915 (Courtesy Museums of World Culture, Stockholm)

**Fig. 5:**
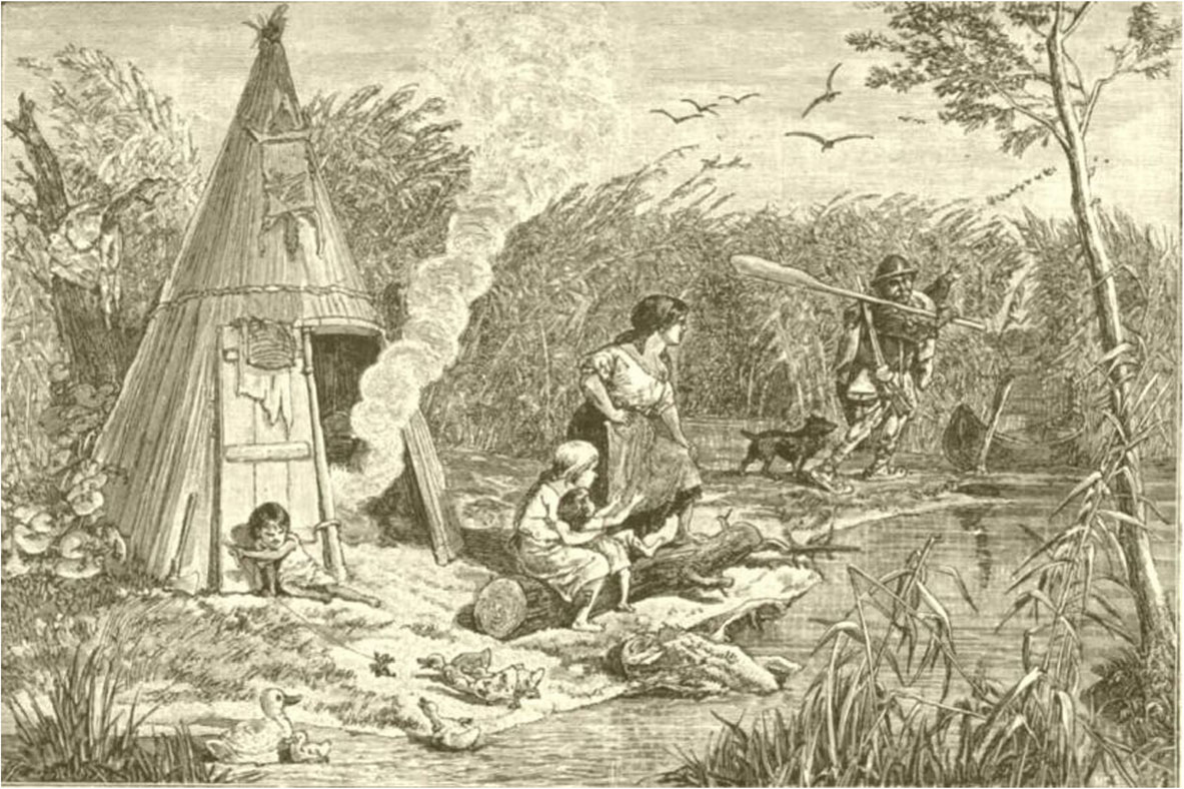
A Pákász family camp in a Hungarian swamp. They were living as fisher-foragers on the marsh-land of the Nagy-Sárrét region (From O. Herman, A Magyar halászat könyve, 1887)

Fisher farmers are found along rivers and lakes all over Europe; there are some interesting minor ethnic groups in the east and south-east where fishing has been very important for their economy. The Onega Vepsians combined fishing with some agriculture [83]. For Vepsians who lived on lakeshores and rivers, fishing was a major form of subsistence [61]. Lake Onega (Karelia, N W Russia) provided them with 40 species, including salmon, trout, smelt and many cyprinids. Lake Peipsi on the Estonian-Russian border area has been important for fishing. There are still ‘Old Believers’ in a few villages continuing the tradition [84]. Evidence of the evolution of fishing traditions has been found from the fish bone data recovered in excavations close to the lake at Pskov and Kamno (spanning the fourth to eighteenth centuries) together with documentary evidence from more recent times. Pike, perch and bream were the predominant species but their size decreased over time and other smaller species became more prominent. This has been attributed primarily to changes in fishing practices with increasing use of nets [85]. The Lipovans (a sect of Russian Orthodox Christians fleeing persecution in the seventeenth century) formed fishing communities in the rich fishing waters of the Donau delta in the Romanian-Bulgarian borderland [86]. On the Hungarian plain, in the nineteenth century, marshes were still exploited for gathering and fishing by the Pákász. They used simple implements, catching fish by hand, used creels and made fish-poison from plants. When the swamps were drained at the end of the century, the fishing economy and the Pákász culture disappeared. The fieldwork of Ottó Herman and Béla Gunda is a source of archival ethnographic data and may provide further information of ethnobiological interest [87, 88]. There are many papers dealing with peasant fisheries in Russia, for instance among the Ural Cossacks settled by the Ural River [89] (Figs. 4 and 5).

Further west, documents from the beginning of our period demonstrate the enduring importance of freshwater fishing in some inland areas far from the coast. Hoffmann [90] made a study of early fishing tracts, two in particular are of interest here. The ‘Heidelberg Booklet’ dated around 1493 and added to over the years, refers to fishing in the Upper Rhine with detailed seasonal advice on a variety of species, the use of baits, traps, lines and nets. Other documentary evidence shows both commercial and subsistence fisheries operating in both private and public waters. A second tract, thirteen pages of manuscript, the ‘Tegernsee Fishing Advice’, was intended for use by the cellarer of Tegernsee Abbey in Bavaria, where fish formed a vital part in the dietary restrictions of the Christian calendar. The precise date is unclear but lies within the late fifteenth and early sixteenth centuries and is also detailed on fish and fishing methods, the best bait and on making artificial flies. In this document, Hoffmann sees the oral tradition of peasant fisheries put into text.

Archaeology and historical documents regarding the freshwater fisheries in northern and eastern Switzerland were integrated in a review of twenty Medieval and Early Modern sites, all situated close to a river or lake [8]. The majority of bone assemblages predate our period but some fit within the time line. The fish assemblages all reflect freshwater fishing; perch and pike were both most numerous and common, marine fish are incidental, largely herring. Salmonids (mostly trout and grayling) and cyprinids were also present. At all sites, many of the fish were small, under 10 cm, both young fish and small species. Larger fish, such as pike and barbel, have been linked to higher status sites, clerical or aristocratic. Documentary data show local fisheries were important supplying the immediate areas; professional fishermen were employed, fishing seasons were limited and setting a minimum size for fish permitted to be caught was designed to protect young stock. In practice, as the bones show, many small fish were caught including immature individuals, possibly a by-catch in a fine meshed net and thought to supply local demand. These could also have been caught by small-scale artisan fishermen. On a commercial scale, the uniform size of larger fish at some lakeside sites is thought to be evidence for a fishery in the spawning season and these fish were marketed regionally. This study shows a correspondence of data between bone assemblages and documents for freshwater fisheries in this area. In contrast, a review of bone assemblages in Flanders indicated a decline through the medieval period in freshwater fish compared to marine at certain ‘urban’ sites such as Ghent. This is attributed to increases in water pollution, blacked waterways and over fishing and a growing demand inland for marine fish inland [91].

In England, a more specialised and traditional fishery is still prosecuted for Atlantic salmon on the River Severn. Fishermen use lave nets, a hand held V-shaped net with a wooden frame, erected ‘fixed engines’ such as putcher (long funnel-shaped traps made of willow and hazel fixed in rows two deep across the river) and stake nets [92]. Similar shaped wicker traps were used singly for eels in the Severn and other rivers across England, particularly in wetland areas such as the Cambridgeshire Fens, where in the early twenty-first century the last eel fisherman using traditional methods retired; he could neither make a living with depleting eel numbers nor find a successor [9]. His family ties with fishing in the area went back to at least the late fifteenth century bringing a long association and generational sharing of knowledge to a close. Ely is a city in the Fens named after the eel, emphasising their former abundance in the area. The wicker traps used for salmon and eel have been ubiquitous in design and material across Europe through time and space, and were also used on the north west coast of North America by the indigenous peoples [25].

Estuarine fishing is well exemplified by the age-old fisheries of the Thames Estuary; ‘whitebait’ is largely composed of young herring and sprats; they shoal together in their juvenile stage. They entered the estuary in large numbers and were the object of a traditional fishery. These small fish rolled in flour and fried, were particularly popular in the riverside London taverns of the eighteenth and nineteenth century. Another small fish that was a traditional fishery in the Thames and other estuaries was the smelt, a seasonal treat and said to taste of cucumber. They enter freshwater in winter to spawn in spring and were netted in large numbers by local fishermen, though sometimes so abundant that their price was very low [93]. Nowadays, smelt numbers, together with salmon and eel, are much depleted through pollution and blocked waterways. Eels were once so numerous they were currency for rent payments, now the rare tiny ‘glass eels’ are part of a lucrative black market, often smuggled through airports in ingenious ways. The inexpensive ‘glass eels’ seen in seafood packets today are made of surimi, a fish paste used in the making of sushi.

A mark of how embedded freshwater fish were and are in aspects of British culture, especially for anglers, is evident in the names of that uniquely British establishment, the pub or public house. These were often named after fish such as ‘The Trout’, ‘The Coy Carp’, ‘The Salmon Tail’ or, reflecting the popularity of angling, ‘The Anglers Arms’, ‘The Anglers Rest’ and many others on a fishy theme. Many early nineteenth century coarse angling clubs held their meetings in public houses where members were fortified by alcoholic refreshments.

**Fig. 6:**
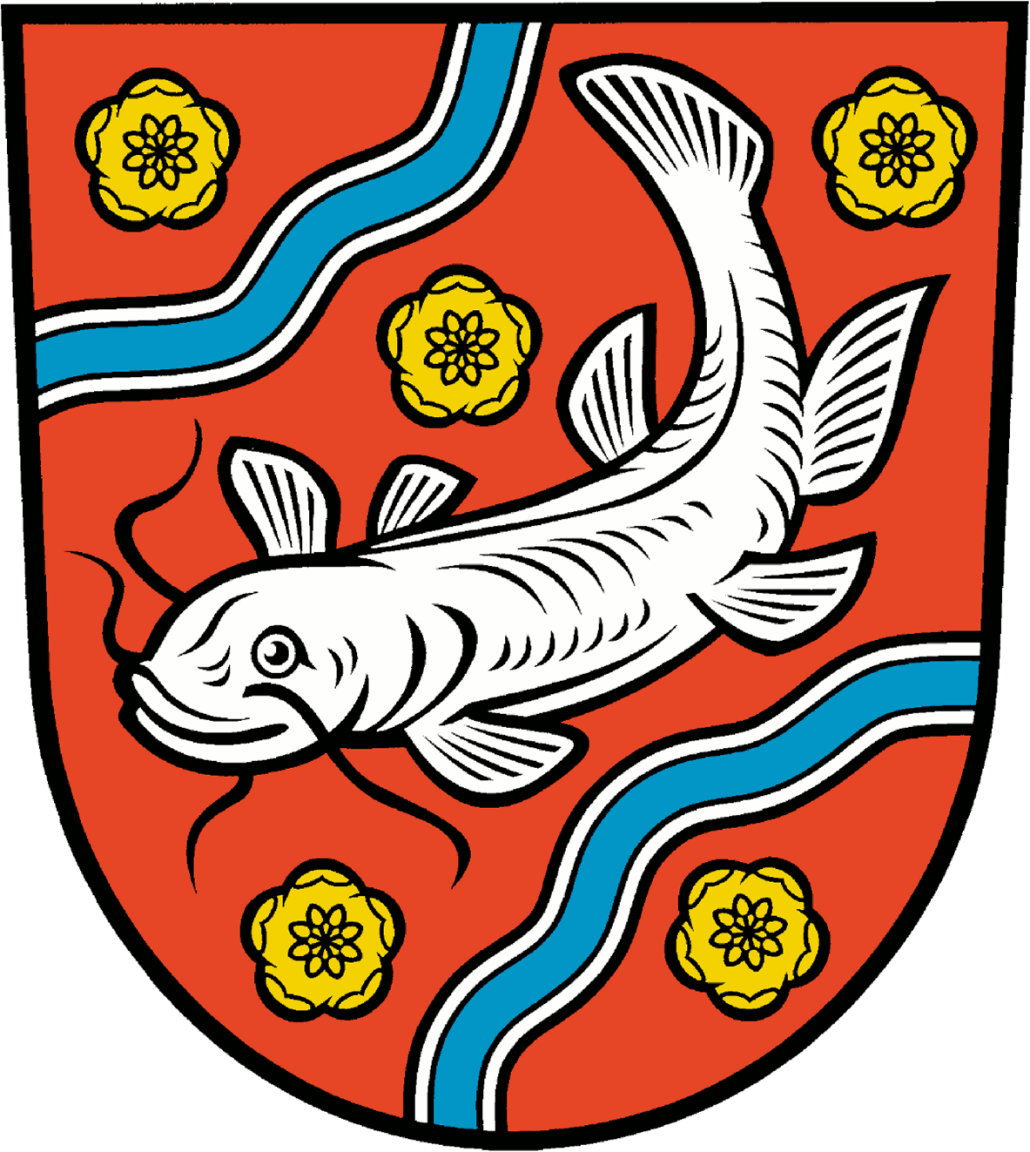
Coat of arms for Oder Welse in Germany. Fish are common on European (including Russian) coats of arms for villages, cities and provinces. Common freshwater fish species depicted are salmon, whitefish, pike, barbel, eel, and lamprey

Freshwater fish are also common motifs in heraldry. They serve as symbols in the coat of arms of many villages, towns and provinces all over Europe, especially where a fishery has brought wealth to the region. The coat of arms for Ely incorporates an arm holding a trident around which is wrapped an eel; for the Oder Welse (Brandenburg, Germany), a wels catfish is depicted (Fig. 6).

#### Local knowledge

Ecologist Fikret Berkes and colleagues characterize local ecological knowledge (LEK) in a procedural perspective as a complex of knowledge, experiences and beliefs. It consists of folk knowledge shared among a specific group of people [94]. This way of reading the biota is based on biological knowledge, personal experiences, ways of classifying the environment, knowledge of lakes and rivers, past observations and a personal conceptual framework for interpreting experiences. All this is conveyed over generations. Language is the most important medium through which local knowledge is communicated and well exemplified by the traditional fisheries described above.

Language contains the analytical tools with which people perceive and manage the landscape and the biological resources. Dialect dictionaries and word collections are therefore an essential source material for the researcher studying popular biology and local knowledge [50]. Furthermore, the linguistic material gives us an opportunity to look back in history [17, 50, 62]. For instance, in northern Scandinavian lakes, the Sami conducted a certain kind of fishing during wintertime called *rackfiske*. It not only confirms the deep knowledge the Sami had of the powan, a whitefish species, the local terminology associated with this way of catching fish seems to contain words that are very ancient and derive from the hypothetical proto-Samic language. It thus bears witness to the great age of this fishing method [75].

Fishing through a hole in the ice, practiced in the circumpolar areas, helped make it possible for humans to settle in the inhospitable Subarctic and Arctic regions [95].

#### Folk classification of fish

According to some ethnobiologists, folk biological classification is one of the most culturally important and most exhaustively lexicalized areas of folk classification among pre-industrial peoples. They reflect their intimate familiarity with the biota [96].

A folk taxonomy is how cultures name, identify and classify living organisms. Anthropologist Brent Berlin’s theories have inspired several ethnobiologists and linguists studying folk biology, including the naming and classification of fish in Europe [97]. Rural communities in Europe developed folk taxonomies for various organisms and grouped them according to use, characteristics, appearance or behaviour [33, 50, 77]. As Lars-Erik Edlund has shown in a fascinating study of whitefish in northern Sweden, fishermen discern several folk taxa of whitefish in one and the same lake, depending on how they were classified by the inhabitants [98]. We have also found interesting folk taxonomies for perch and pike, where they and other taxa were named after their occurrence, size and colour variations. For instance, the pike was named after various spawning periods [50]. About 100 years ago, fishermen along the coast of the Swedish province Hälsingland distinguished between 25 different kinds of Baltic herring, their names indicated the size, colour, spawning locations, etc. [99]. When it comes to the European smelt, the fish is caught during a very limited time giving rise to a series of terms that focus on the circumstances that prevail at the time of the fishing season [31, 77]. Estonian researcher Mari Kendla has researched Estonian local folk categories of fish, which shows that external characteristics of fish are important for classification [100].

#### Folklore and folk religion

Folklore is a part of traditional fisheries; like hunting, fishing is quite uncertain and unpredictable, and thus more susceptible to a poor return than some other economic activities [101]. It was therefore important for a fisherman, through various measures and rituals, to secure his catch, usually at the expense of others. This explains at least in part the rich flora of stories, ceremonies, rituals and customs associated with fishing. In collections of local folk beliefs, we often find fishery folklore is largely characterized by the desire to promote one’s own catch and protect oneself, the boat and the gear from destruction and from others. There are plenty of recorded data in our archives; however, there are few studies that specifically address the folklore of fisheries [102, 103].

This rich vein of folklore, including proverbs, sayings, fish-prayers, taboo observations, songs, stories, etc. deserves further research for our understanding of how people perceive fish. It has for instance been a widespread practice to read signs of fish behaviour or presence to predict the future or weather. We can talk about ichthyomancy (divination by interpreting the appearance, behaviour or entrails of the fish), which is still practiced also within Europe [50]. Along the coasts of Scandinavia and the North Sea, it was common to hang on a string from the ceiling a so-called *weatherfish* or *wind fish*, in order to predict or observe the direction of the wind. Most of them were nearshore fish from the intertidal zone. However, the custom existed also along the Baltic Sea coasts [102, 104].

Fish have played a role in religious folklore connected with the church, rosaries have been found made of fish vertebrae and depicted in religious art, possibly connecting to Christ as fisher. In some areas, unusual large and rare fish, such as sturgeon, were (in the same way as whale bones) displayed in churches, for instances along the Baltic coast [50]. Fish were also used as votive offerings to secure luck for the fishermen [105].

An interesting aspect of the relationship of fisherfolk to their catch is the use of taboo languages, which is well documented when it comes to the Faroese, Norwegian and Shetland context. Martin Martin cites examples from seventeenth century Orkney [50, 63, 106]. However, it is practiced by fishermen elsewhere, including those catching freshwater fish, but it has been very little researched. Per Arvid Säve, in a book from 1888, gives an interesting example from the island of Gotland [107]. The use of noa-names that replace a taboo word is common [50]. The phenomenon has probably been much more common among fishers than the research to date indicates and there is scope for further investigation.

#### Subsistence fishing and its technology

**Fig. 7:**
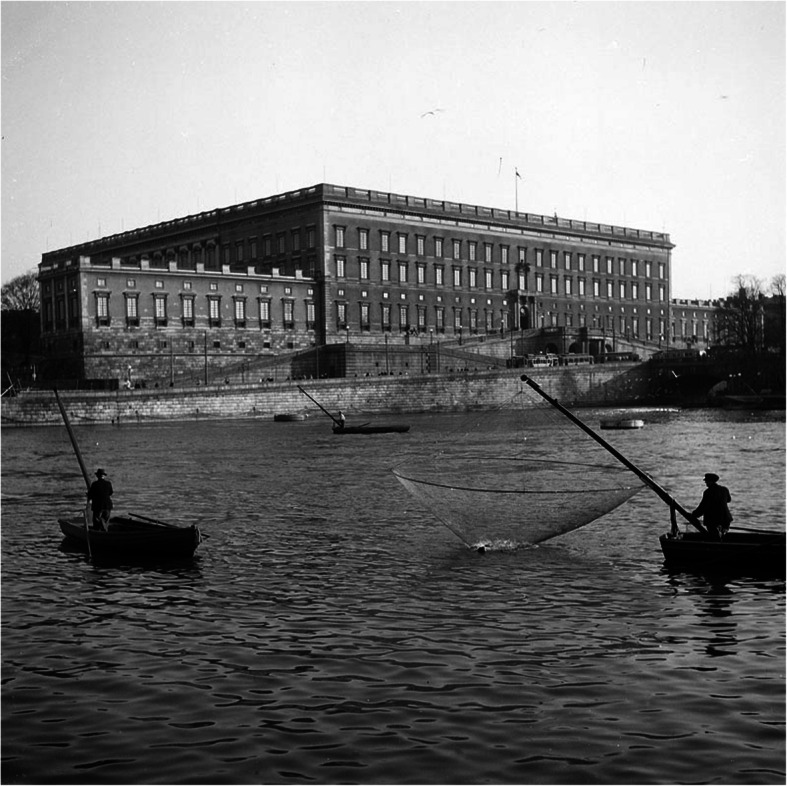
Fishing for smelt in Norrström, Stockholm, in front of the Royal Castle in 1945. This view was well-known to people in the city since medieval times and lasted until 2016, when the last smelt boats were sold (Photo Lennart af Petersens, Courtesy Stockholm City Museum)

Artisanal fishery can be variously defined as small-scale, low technology, low-capital fishing practices undertaken by individual households. This kind of fishing uses techniques practiced over centuries including a variety of nets, traps, harpoons, lines and traditional fishing boats on rivers and lakes (Fig. 7).

These fishing practices may be undertaken for both the local market and for subsistence. It contrasts with large-scale modern commercial fishing in that it is often less wasteful and less stressful on fish populations [108]. Artisan fishers with rights on local waters have a personal investment in preserving fish stocks for future harvesting.

**Fig. 8:**
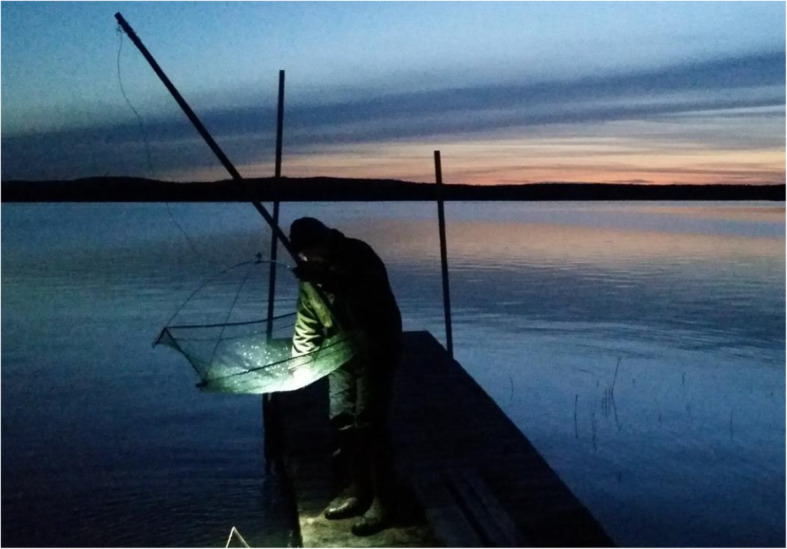
Night fishing with a traditional lift net at Sindret, Värmland, in April 2019. This gear was used only a few kilometres from where Linnaeus made a similar drawing in 1746, c.f. Fig. 9 (Photo Armas Jäppinen)

**Fig. 9:**
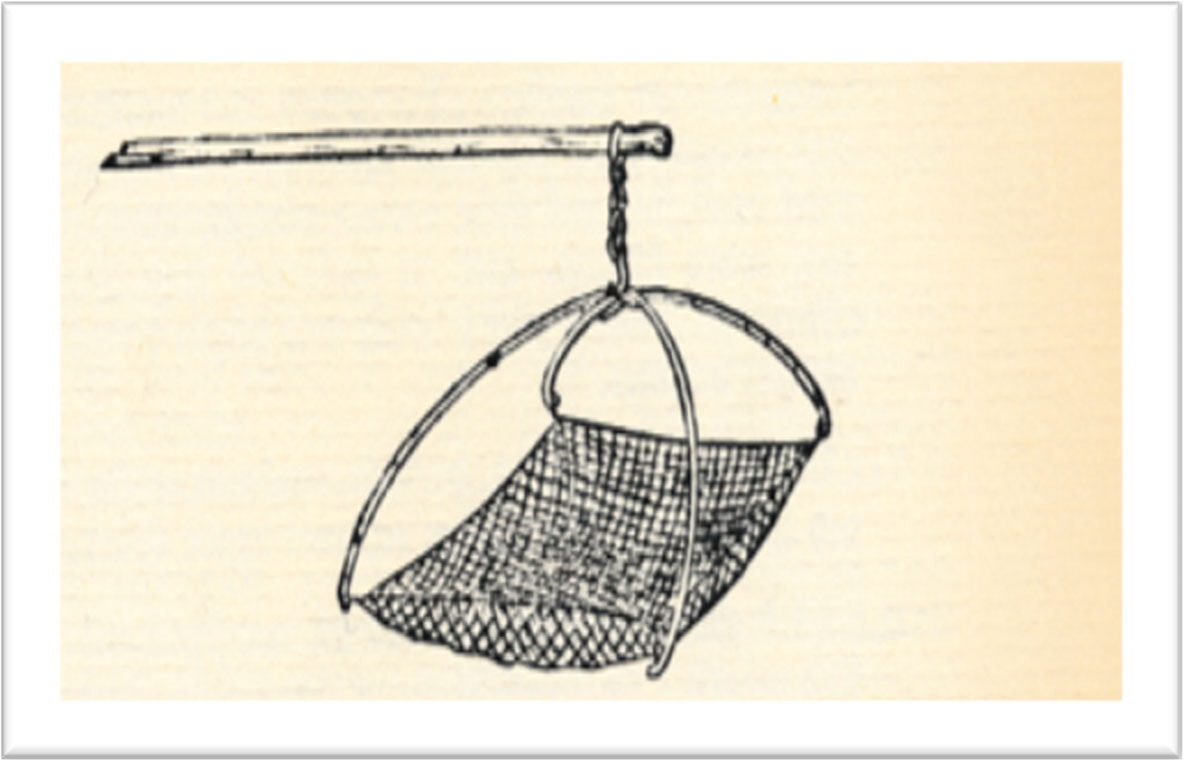
Lift net for smelt fishing observed and sketched by Linnaeus in Persberg, eastern Värmland, in 1746 (Source Carl Linnaeus, Wästgötha Resa, 1747)

There have been a number of studies on traditional fishing gear. A groundbreaking study, on fixed fishing gear in Finland and Russia, was made by the ethnographer, Uuno Taavi Sirelius, at the beginning of the twentieth century [34]. Subsequent works include Andres von Brandt’s *Fish Catching Methods of the World* [39]. In Sweden, studies of fishing gear and local fishing have been carried out by ethnographical researchers such as Carl Gustaf Lekholm, Torsten Jonsson and Nils Nilsson [50, 109]. However, the most interesting data from an ethnoichthyological perspective were gathered by fisheries biologists. They often stayed in the field in close contact with locals and specialised fishermen. With the help of questionnaires, they collected extensive data on fish, fish names and fisheries in different lakes and rivers around Sweden. These include Ivar Arwidsson, Anna Arwidsson, Torsten Ekman, Rudolf Lundberg and Filip Trybom. They published in various fishing magazines, collected fishing gear, photographed and made general inventories of fish stocks and the local people's utilization of these resources [35, 50, 110] (Figs. 8 and 9).

#### Simple fishing methods

**Fig. 10:**
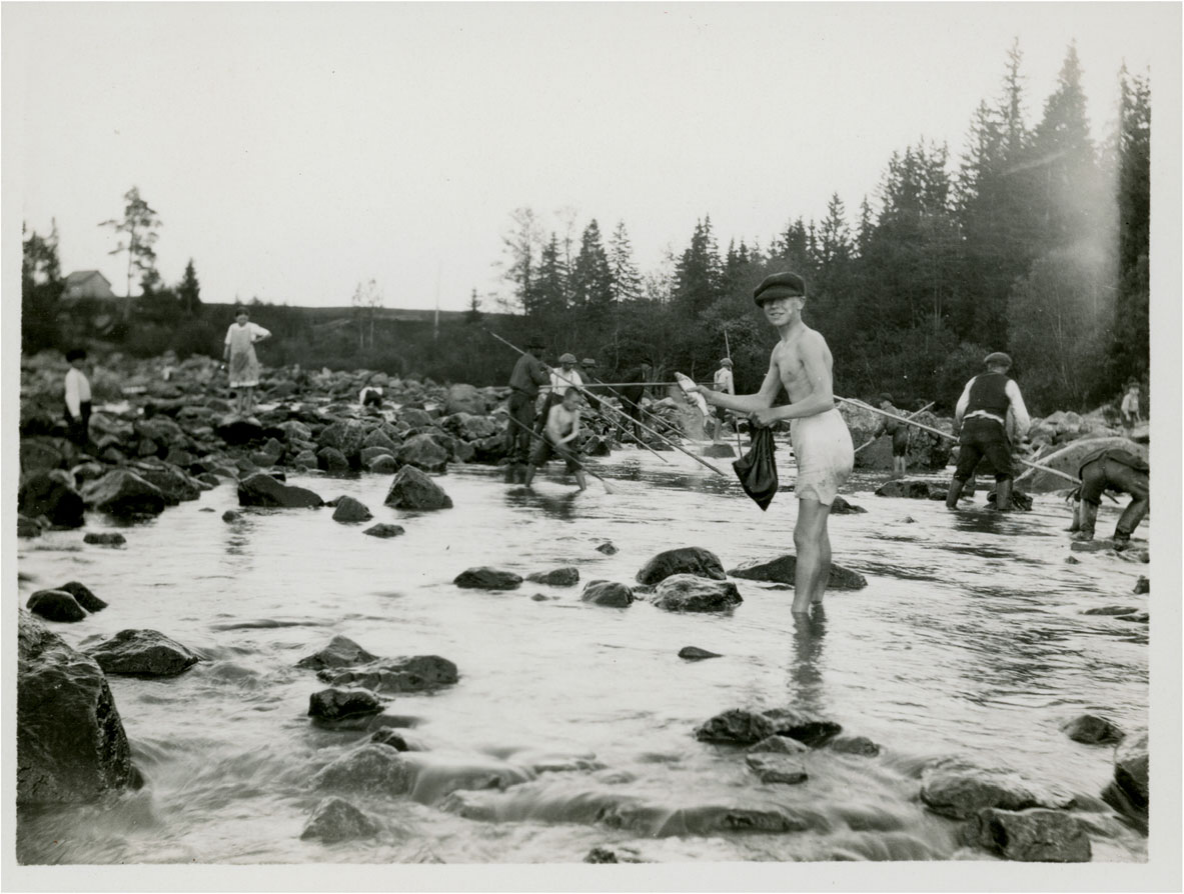
Vimba bream (*Vimba vimba*) captured with nets and by hand in a stream at Strömsberg in Finland 1926 (Photo Curt Segerstråle, The Society of Swedish Literature in Finland, Helsinki)

At the most basic level, collecting fish by hand is very simple and probably the most ancient of all fishing practiced by humans. It is a universal custom that has been recorded from many parts of the world [39]. There are many examples from Sweden, and it has been used until quite recently. Smelt shoals running in the rivers of Värmland can still be very dense and the fish can be harvested by hand in large quantities. According to observations in the 1950s, boys in western Värmland could apparently catch up to 25 kg in this way [31, 111]. Single fish can be caught by ‘tickling’ where the moving of fingers in the water under the fish causes it to rise and with a swift movement gripping the head and tail the fish can be lifted out of the water [112]. This method has often been used on trout, hence often referred to as ‘trout tickling’, as well as other fish. Catching pike and Arctic char from a boat or land with the help of a snare made of brass wire or other materials has been common all over Sweden. Fishing by hand is of course also known elsewhere in Europe, although seldom discussed in the literature [35, 36, 50] (Fig. 10).

#### Poison and stunning

Fish poisoning is an interesting method of fishing practiced especially in Asia and South America [39, 113]. The purpose of poisoning is to stun or stupefy the fish so they can be easily caught. However, plant-based poisons are also used in the Balkan Peninsula and parts of Eastern Europe, according to studies by Bosnian, Polish and Hungarian ethnologists [36, 87, 114, 115]. Plant-based poisons were used in northern Europe and from Central Europe where it is well documented that seeds from mullein were used for stunning fish [114–116]. There are also a few records of its use in Sweden [50]. In France, chloroform was used by poachers to stun salmon in the early twentieth century as fish prices rose during World War I; dynamite was another tool to flush out salmon for easy capture. In Ireland, the crushed roots of Irish spurge was an effective stupefier, while in Spain an old sock filled with chloride dipped the water would soon cause salmon to flee into the poacher’s net [117]. Spain has a long tradition of using plant-based poisons in fishing, as demonstrated by a royal edict in 1255 banning their use. Successive legislation indicates that this was a common, though illegal practice, based on species such as the Euphorbias, mullein and henbane as well as walnut, flax-leaved daphne, common leadwort and villious deadly carrot*.* Similar practices are found in other southern European countries such as Italy, Sardinia and Portugal [116].

Fish biologists have used rotenone in order to kill off fish populations from small lakes. It is a non-selective fish poison, usually made from the plant known as the lancepod native to South America [111]. Biologists also use electricity for shocking fish; however, it could also be used for illegal fishing. Use of explosives for fishing is a problem in many parts of the world, including Europe. In Sweden, although illegal, it was still used in the late 1940s and is still used among poachers in, for instance, the southern Balkan Pensinsula [39, 50].

**Fig. 11:**
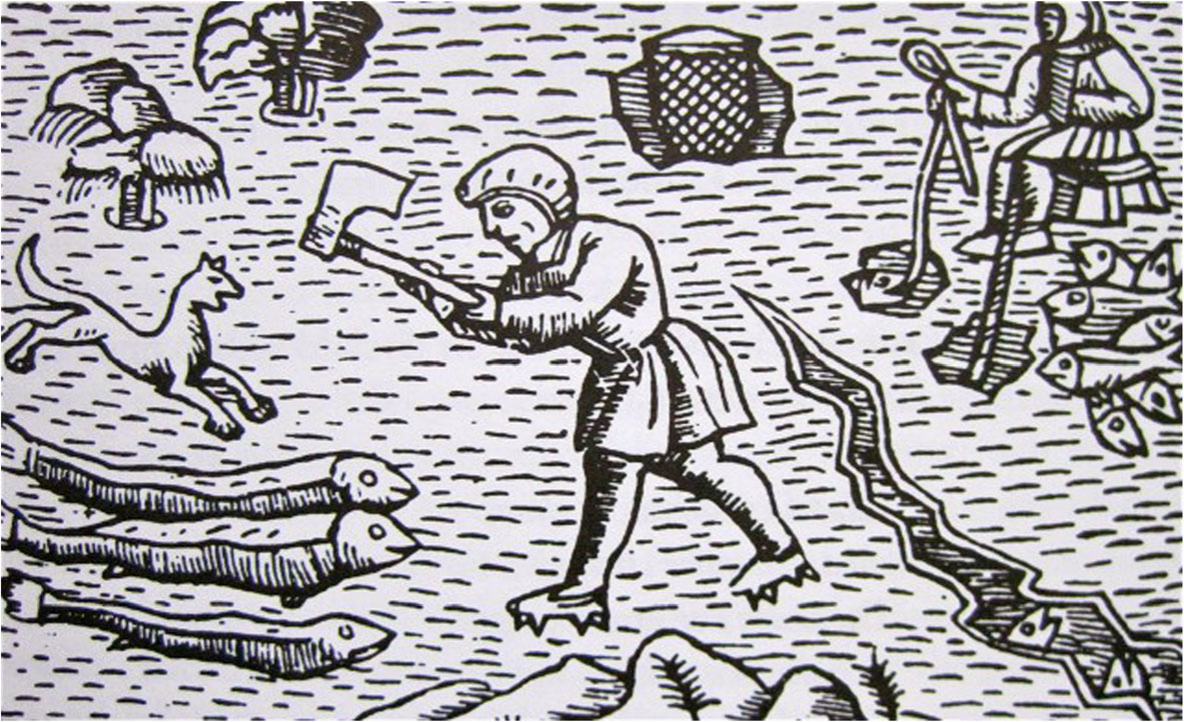
Stunning burbot, *Lota lota*, through the thin ice with an axe (From Olaus Magnus, Historia de gentibus septemtrionalibus, 1555)

**Fig. 12:**
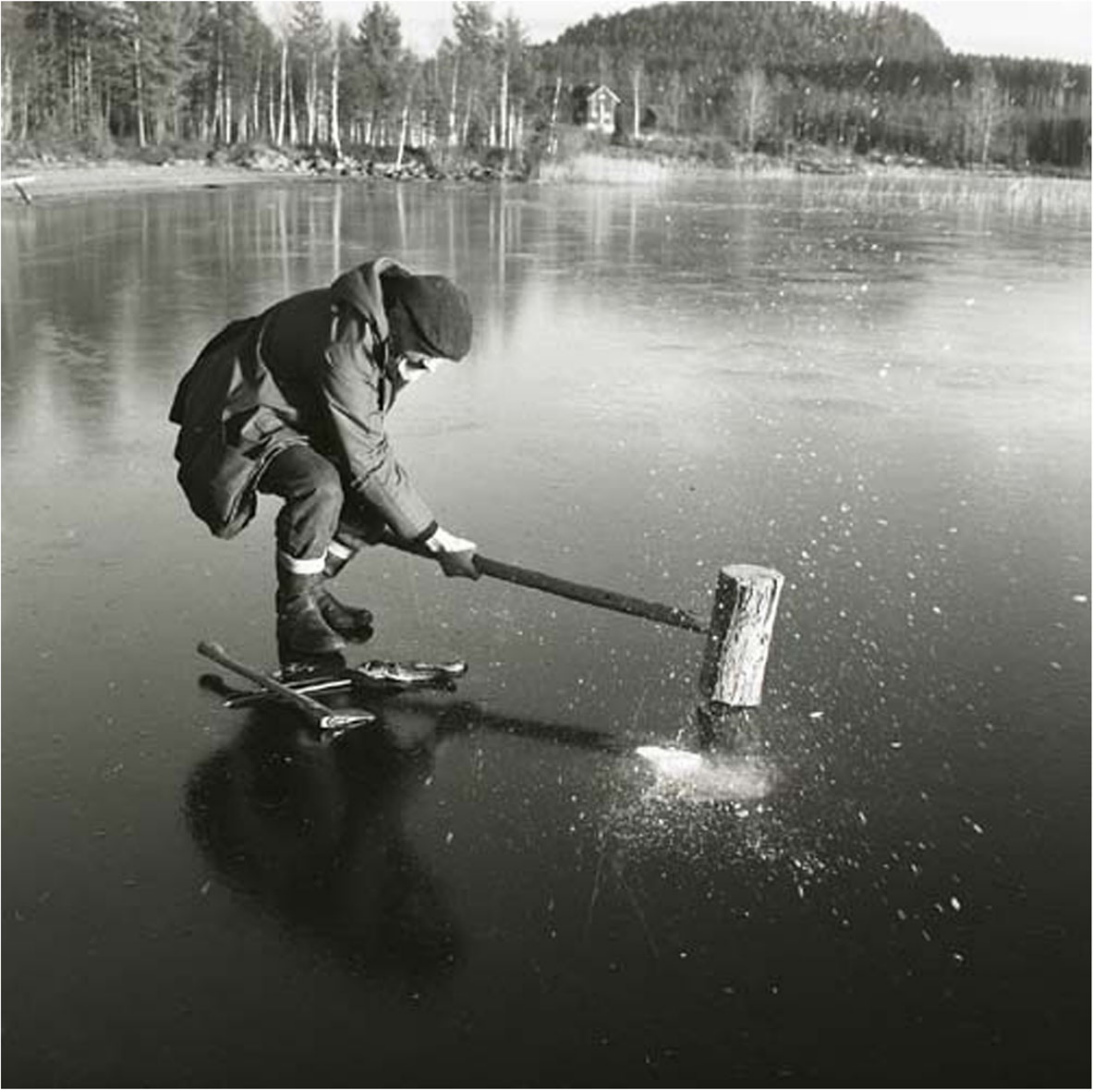
Some old-style fishing techniques have survived until today. Here is a man stunning burbot, through the thin ice with a bludgeon, c.f. Fig. 11 (Photo Hilding Mickelsson 1972, Hälsinglands Museum, CC-BY-NC)

Another well-known method of stunning fish in North and East Europe, especially burbot and pike and mentioned by Olaus Magnus in 1555, is to hit them on the head with a bludgeon or large hammer. This has been practiced all over Sweden and was still known a few decades ago [36, 39, 84, 87, 107, 118] (Figs. 11 and 12).

#### Kleptoparasitism and fishing with trained animals

There are other fishing methods seldom dealt with in the literature. In a variety of ways, humans have long depended on animals in their foraging activities. Simply stealing what you can eat from other species is a prehistoric habit that continues to this day (e.g. to gather eggs and take nestlings from birds’ nests for food, nuts and seeds from vole borrows, or collecting honey from wild bees and bumblebees). These are practices with a long tradition [119]. Kleptoparasitism, the act of stealing food from other species, has been a common strategy in the human search for sustenance. Taking advantage of what other species have already gathered is an easy way to obtain food. Indeed, stealing prey from predators was probably practised from earliest times. It was, for instance, a practice among coastal dwellers to rob fish from various bird species. In Sweden and Finland, coastal people practised kleptoparasitism on the nests of white-tailed eagles and ospreys. The method was simple. First, the nestlings were chained to the nest when they were beginning to leave it. Daily, the nest could be emptied of fish that the parent birds had brought home to their young. Tying the nestlings’ cloacae with string could make this more efficient preventing them from eating all the fish the adult birds had brought to the nest [120]. The same practice is described for the Pákász in the Hungarian marshes, who also robbed the nests of white-tailed eagles [121] (Table 2).

**Table 2:** Other organisms mentioned in the text

Black throated diver	*Gavia arctica* (L., 1758)
Red-throated diver	*Gavia stellata* (Pontoppidan, 1763)
Great crested grebe	*Podiceps cristatus* L., (L., 1758)
Cormorant	*Phalacrocorax carbo* (L., 1758)
Red-breasted merganser	*Mergus serrator* (L., 1758)
Goosander	*Mergus merganser* (L., 1758)
White-tailed eagle	*Haliaeetus albicilla* (L., 1758)
Osprey	*Pandion haliaetus* (L., 1758)
Eurasian otter	*Lutra lutra* (L., 1758)
Dog	*Canis familiaris* L, 1758
European Crayfish	*Astacus astacus* (L., 1758)
White-clawed crayfish	*Austropotamobius pallipes* (Lereboullet, 1858)
American signal crayfish	*Pacifastacus leniusculus* (Dana, 1852)
Medicinal leech	*Hirudo* sp.
Swan mussel	*Anodonta cygnea* (L., 1758)
Thick shelled river mussel	*Unio crassus* (Philipsson, 1788)
Fresh-water pearl mussel	*Margaritifera margaritifera* (L., 1758)
Mullein	*Verbascum thapsus* (L.)
Irish spurge	*Euphorbia hyberna* (L.)
Henbane	*Hyoscyamus niger* (L.)
Walnut	*Juglans regia* (L.)
Flax-leaved daphne	*Daphne gnidium* (L.)
Common leadwort	*Plumbago europaea* (L.)
Villious deadly carrot	*Thapsia villosa* (L.)
Lancepod	*Lonchocarpus* sp.
Common reed	*Phragmites australis* (Cav.) Trin. ex Steud.
Club-rush	*Schoenoplectus lacustris* (L.) Pallas

Source GBIF | Global Biodiversity Information Facility (www.gbif.org)

People have used various species to assist them in hunting and gathering activities [122]. Some mammal species have been used for fishing. The Ainu of northern Japan and Sakhalin trained their dogs on command to swim in packs, frightening fish into shallow water, where they were easily caught [39]. Fishermen in coastal Portugal similarly taught dogs of the local breed Cão de Água to herd fish into fishermen’s nets and to retrieve lost or broken tackle [39, 123]. Gunda cites a few examples of dogs used for fishing from Rétköz in the Carpathian region and from Hortobágy on the Hungarian plain [121].

The use of tame cormorants to assist in fisheries is a well-known example of animal helpers in the ethnographic literature. Cormorant fishing has been especially common in Japan and in parts of China [122, 123]. Some examples of cormorant fishing from Europe (England, France and Hungary) are also known, although it was never a widespread practice there [124]. The earliest reliable source stems from Justus Joseph Scaliger in 1557, reporting cormorant fishing from Venice [122, 124].

A similar practice to the use of cormorants took advantage of diving seabirds to drive fish shoals into nets or traps. Their ability to concentrate significant quantities of fish quickly may have been of primary importance, for instance diving birds were used to drive fish shoals at Doiran Lake in southern Macedonia. Working groups of fishing birds were formed, including red-breasted merganser, great crested grebe, cormorants and black and red-throated divers (loons). The wings of some of these birds were clipped and they were used to drive fish into fenced areas [125]. There are also a few examples of this in Sweden and Finland. In 1749, the Reverend Johan Ilström described how the peasantry in the Kalmar Strait took advantage of the gathering of goosanders in the shallow bays during the autumn. Goosanders appear in large flocks and fish collectively by sitting on the water in rows and scaring the fish into shallow bays. Local people noticed this behaviour and constructed special traps under the surface of the water where the birds appeared in the autumn. The fish took refuge in these traps to escape the birds and the locals collected the fish. According to Ilström, it was a very profitable way of fishing. This method of fishing with the help of goosanders continued to be used along the Baltic Sea coast into the nineteenth century [122].

**Fig. 13:**
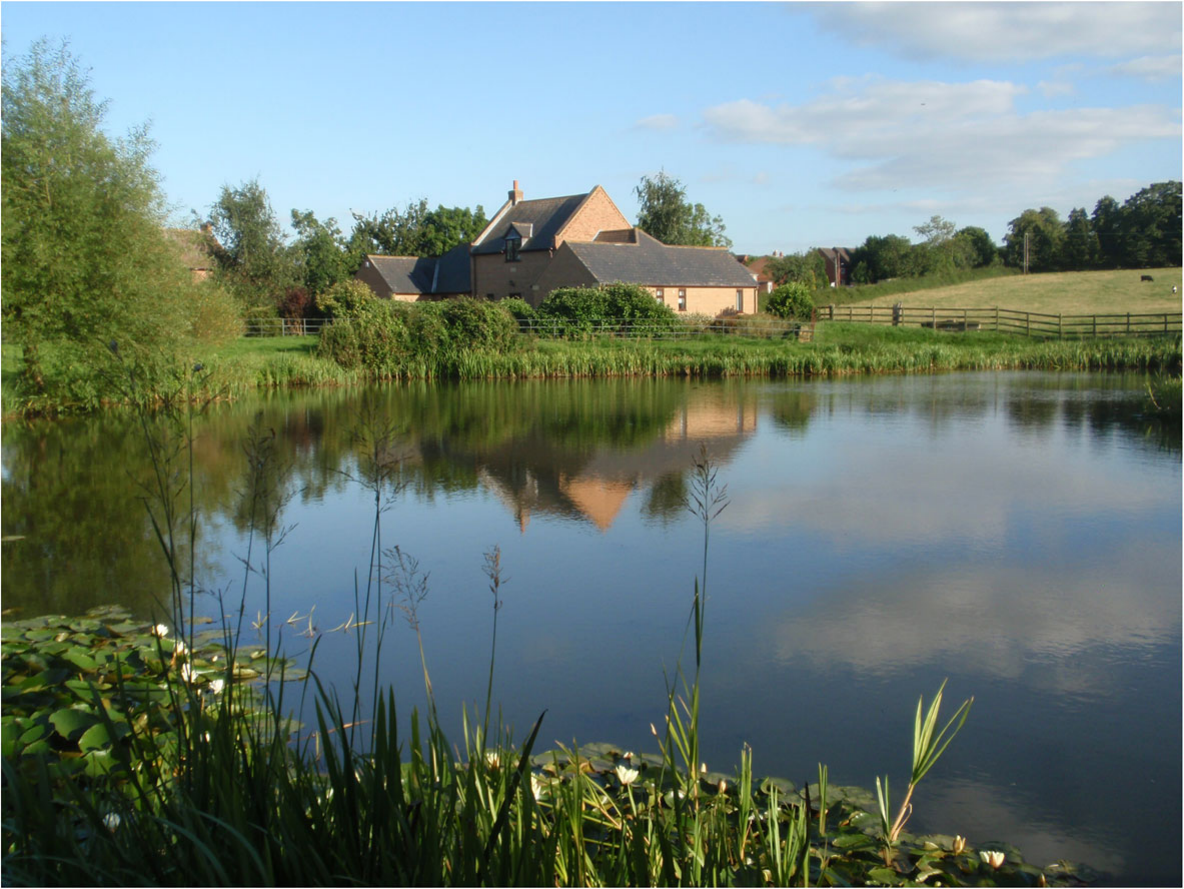
An old fishpond of medieval origin still stocked with fish at the Manor House, Long Clawson, Leicestershire, England (Photo Nicholas Redman, 2012)

Fishing with trained otters has been carried out for centuries in Asia and Europe. There are several species of otter in Eurasia, but only two seem to have been employed to assist with fishing. The Eurasian otter has been trained for the purpose. A tame otter soon comes to know its owner and follows them about like a dog. Fishing with otters has been practised in Europe since the late Middle Ages. According to late medieval zoologists such as Thomas of Cantimpré (*Liber de natura rerum*, 1225–41), Vincent de Beauvais (*Speculum Naturale*, 1244) and Albertus Magnus (*De Animalibus*, mid thirteenth century), otters were trained to fish in central and western Europe. They were taught to dive after the fish and drive them into fishermen’s nets; this technique is probably most suitable for catching salmon. The English author and noted angler Izaak Walton (1594–1683) also cites this custom in his famous *The Compleat Angler* (1653), reprinted many times and translated into many languages [122, 126]. In a discussion on otter hunting, once a popular pastime in England, where the wild otter was seen as the enemy of the angler and disliked by fish farmers today, Walton wants the hunter to keep one otter pup for him. He knows of a gentleman in Leicestershire ‘who hath not only made her tame, but to catch fish and do many things of much pleasure’. The otter was muzzled to prevent it from eating fish, and was fastened by a leash to its master. Fishing with otters is also mentioned in the Hebridean Islands, off the west coast of Scotland, by the Scottish author Martin Martin around 1695. The otters were ‘trained to go a Fishing, and fetch several sorts of Fish home to their Masters’. We have several more examples from England, recorded for instance by the Reverend John George Wood (1827–1889). Frédéric de Tschudi provides a Swiss example, claiming that otters could be trained to go into the water on command and bring back fish to their masters. There are also data from the Kerzhenets River in Russia and many examples from Poland [127] and likely from elsewhere in Europe (Fig. 13).

It seems also to have been a widespread practice among Sami fishermen to use otters for fishing until recently, according to a study from Lule Lappmark. In winter, when it was hard to catch fish using snares or nets under the ice, the otter was especially useful, as it could be released into open holes in the ice. To get an otter for training, the Sami hunters searched for them during the spring when they gave birth to pups in their holts. Male pups were preferred to females, which were left with their parents. Once you have taken care of an otter pup you are then obliged to keep it for life. In the beginning, the pup was kept in a cage made of willow branches and fed with small live fish. The pup was easily tamed and could be released from the cage to live in the hut or tent together with its owner. They were kept separately from dogs. A tame otter can learn to fish by itself, but preferably the pup was trained by an adult tame otter. The otters were named and controlled using whistles and verbal commands. Not only did it catch fish for its owner but it could also show where fish were located in the streams and lakes and drive the shoals towards land. The Sami in northern Scandinavia considered the otter as a species that should not be hunted and those Sami relying on fishing very often tamed otters to assist them in the fishery [122, 127].

#### Traditional methods of preserving fish

As a highly perishable, protein-rich food, fish must be prepared properly for storage to ensure it will last for later consumption. Drying, fermentation, salting and smoking were common, time-honoured methods of storage before refrigeration and freezing became standard. The oldest way of preserving fish is by air-drying, which has been widely practiced in Europe [31, 50, 83, 128]. To be successful, the moisture level must be sufficiently low to prevent bacterial growth; the most famous being stockfish prepared from cod on the Lofoten Islands. White, low-fat fish are most suitable for drying; oily fish will quickly become rancid. Freshwater fish have also been dried including pike, smelt and wels. Norsander, in a study of fish eaten in rural Sweden in Småland in the late nineteenth century, found that although herring, cod and stockfish (dried and sometimes salted cod or ling) were the majority of fish eaten, there were also some lake fish; bream, roach and pike and these were dried [129]. Salting was often used in combination with drying where climatic conditions were too damp for drying alone. Salmon was salted, powdered (dusted with salt) and pickled in large quantities with Scottish and ‘Newcastle’ salmon (which was actually caught in the Tweed then salted and dried or boiled and pickled) bound for London or export in the eighteenth century. These forms of stored salmon were common and apocryphal accounts cite discontent with the frequency with which it was served [9]. Salted dried fish (bream, roach, ruffe, smelt, vimba) are still popular as beer or vodka snack in the Baltic States and Russia [50].

Other methods include fermentation, smoking and pickling. Fermentation is an ancient practice that is still in use in Norway (Norwegian *rakfisk*, made of trout, Arctic char or grayling) and in Sweden (Swedish *surströmming*, made of Baltic herring). Fermented roach (Swedish *surmört*) has been a staple food in northern Scandinavia, although it has now almost vanished completely. In the past, whitefish and other fresh water species were also fermented. Sea fish are fermented in the Faroes and in Iceland [50, 130, 131], while in Kamchatka salmon were buried in the ground fermenting into a ‘cheese’ [76].

*Gravlax* is nowadays a popular Scandinavian dish made of salmon that is cured using salt, sugar and dill. Although the name is ancient, the contemporary dish has nothing to do with the way it used to be prepared in the early twentieth century. The old method is known from medieval times, when the lightly salted fish were placed in pits dressed with fir twigs or birch bark. Lactobacteria preserved the fish, in this way they could save on the use of expensive salt. The evolution of some ‘ancient practices’ invites some caution when citing language as evidence of longevity as many ‘artisan’ products have been adapted to modern tastes [132].

The European eel has a high oil content and is often smoked and pickled; smoked eel has been revived in England as an artisan quality product. Jellied eels (eels boiled in a spicy stock that sets into jelly on cooling) were a traditional food among inhabitants of the east part of London. The tiny ‘elvers’ were once caught in their thousands and local people in Somerset (England) used to season and cook these fish pressing them into a cake; once cold, it could be cut into slices; the decline of the eel makes this dish a distant memory [133].

Potting was another way of preserving fish and other meats prior to chilling and freezing. Lampreys were potted in Worcester, which lies on the river Severn, where the river lamprey used to be very common and so delicious that legend records a surfeit led to the death of two kings of England. Lake Windermere in the Lake District of England is home to Arctic char, a relative of the trout; land locked populations remain in a few glacial lakes. Initially preserved in pies, in the later seventeenth century char were potted; the cleaned, spiced fish were packed in a dish, sealed with butter and cooked. The decorated ceramic pots became collectors pieces much sought after today. By the mid nineteenth century, overfishing left its mark and despite fishing restrictions the stocks are still low and potted char are in very limited supply [134].

Smoking is another way of preserving fish. Fishermen constructed smoke houses of wood, stone or other material. The smoked fish could be kept for several months. It is still a common practice and smoked fish are popular within the trade; besides eel, herring sold as *buckling* (German *Bückling*, Swedish *böckling*), *kippers*, *Yarmouth bloater*, etc., whitefish and salmon, most fresh-water fish can be cold or hot-smoked, which can observed for sale in fish markets in Finland, the Baltic States, Russia and elsewhere [30, 135, 136]. High-quality fish, often salmon and trout, are smoked over different woods, peat and other additions such as berries, whisky and herbs to impart different flavours to the fish. They command much higher prices than the chemically enhanced varieties [136].

#### Fish for food

Freshwater fish were a source of fresh protein for many landlocked communities from time immemorial. Bulked up with vegetables, bread and later potatoes, most species were edible, with small fish made into soups and stews flavoured with wild herbs. Poor rural communities, apart from a few commentaries by early travellers, diarists and proto-ethnologists gathering data, are fairly silent in the record before the nineteenth century and we must rely on the bone evidence from archaeological excavation, which indicates the species but not the cooking method [9]. The wealthy, by contrast, have left a much richer documentary legacy for the preparation and serving of freshwater fish, either from their own ponds or purchased. Household accounts, cookery books, estate records, court cases and descriptions of entertainment and meals by guests provide insights into both management and consumption of freshwater fish.

They were evidently important in French noble houses of the seventeenth century as shown by the recipes for pottage, ragout, roasting, stewing and grilling of carp, tench, barbel, bream, dace, chub, salmon, burbot, eel and many other fish from freshwaters listed by Varenne, a master chef to the Marquis d'Uxelles at his chateau in Bourgogne [137]. Indeed, many noble houses in France, England and across Europe not only valued freshwater fish on the table but incorporated them into their coats of arms, often as a play on the family name, or reflecting the source of their wealth [138].

The demand for a variety of freshwater fish in France continued through the ‘Ancien Régime’ (late fifteenth–late eighteenth centuries) as demonstrated through the supply of live fish to Paris from managed ponds, some very large and some distance from the capital; the fish were transported in live wells along rivers and canals to the Paris markets [139].

Fish used in traditional Balkan recipes include the asp, bleak, carp, nase (cyprinids), as well as eel, perch, pike-perch, trout and wels; all were baked, grilled or fried [140]. Similarly in Poland, a strict adherence to catholic fasting days in the Medieval period and difficulties of delivery from the coast raised the status of freshwater fish, which have remained more popular in the east than other parts of Europe. However, here too figures show falling consumption as the globalisation of food, including fish, slowly erodes traditional practices such as the Christmas carp, bought whole, live and kept in the bath until needed. Fillets especially from marine fish are now preferred by younger generations, especially in the growing urban centres.

The global transformation of the food system with increased access to marine fish products (both fresh and frozen from distant waters), which both caters to and influences changing tastes, has strongly influenced the decline in demand for freshwater fish in Europe [141]. The destruction and pollution of rivers and lakes, with toxic substances from households as well as industry compromising water supplies (for example unwanted medical supplies flushed into domestic waste systems), have also given rise to discussions as to whether these fish are safe to eat. As fish stocks dwindle, there is doubt that local fishing is sustainable in many parts of Europe. All these factors contribute to the erosion of many local foodstuffs [9, 142].

In order to re-awaken or sustain the interest in the fish, some localities have held special gastrofestivals. Holding these regional food festivals to promote tourism and local food products is a global trend [143]. At Ely in the Cambridgeshire Fens in eastern England, the city named after the eel holds an annual festival devoted to the eel with parades and food stalls. One of the oldest is probably the White Fish Festival in Kukkola, in the border area between Finland and Sweden [144]. Vendace Day in Bengtsfors, Dalsland, southern Sweden is another example of a local fish festival. Since 1990, Norway has celebrated a rakfisk festival in Valdres on the first Saturday in November. The most well-established gastrofestival in the Nordic region is the annual Baltic Herring Fair (*Silakkamarkkinat*), held every October in Helsinki, where other fish products, such as vendace (a freshwater whitefish), are also promoted. Smelt festivals are part of this trend, and have been held in the Swedish cities of Mariestad and Arboga. In Europe, festivals of this kind are held around the Baltic Sea. In Palanga in Lithuania, the annual smelt festival takes place in January. It is known as the Palangos Stinto. In Finland for some years, the city of Paltamo has held the Norssikarnevaale (‘Smelt Carnival’). Considerable funds were invested locally in an attempt to develop commercial smelt fishing. Now, however, the carnival seems to be fading away, and its festive nature has largely been lost. In 2015, it consisted mostly of onlookers watching the smelt fishing [31, 77]. In Saint Petersburg, a smelt festival (Russian: *Prazdnik koryuški*) has been held since 2002 in the month of May at the time of the fish’s annual spawning run in the River Neva [145]. Similar festivals are arranged in North America [31]. If we include sea food, there are many gastrofestivals in Europe, the most noteworthy is probably the Oostduinkerke shrimp festival in Belgium, where local fishermen ride on horses to catch the shrimps. In 2013, this way of fishing was inscribed in UNESCO’s list of of the Intangible Cultural Heritage of Humanity [146].

There are also initiatives within the Slow Food movement to increase interest for local fish products, Slow Fish, with food fairs. Is it possible to influence the market and increase the demand for fresh water fish again? Is there a future for small-scale fishers in lakes and rivers within Europe? Research on the importance of food festivals and other attempts to popularize freshwater fish as food are as yet few.

#### Other uses of fish

Non-culinary uses of fish are varied; isinglass was made from sturgeon, carp and wels. Bleak scales were made into ‘essence d'orient’, a coating for artificial pearls, though several thousand fish were needed to produce 100 g [50, 147]. The method was popular in Eastern Europe and France, with the descaled fish sold for food. Fish skin has had many uses; salmon and eel ‘leather’ is well known and still used today by some artisan, quality craft workers for accessories, such as belts, wallets and handbags. Since many exotic species are no longer legal in the fur and leather trade, fish skin may become more in demand [50].

Skin and scales have been used as clarifiers for beer and in glue manufacture. Traditional uses of eel skin in agricultural and other rural contexts for straps and containers in preindustrial times have been described by Berg [148]. Other species include burbot, salmon and a variety of marine fish. A piece of dried pike skin was until recently used to clear coffee in Sweden [50].

Farmers and fisher-peasants have a long tradition of using ‘trash fish’ (fish considered low grade or over abundant and often small) as fertilizers on fields [50, 149–151]. Such superfluous fish have also been used as animal feed for cattle, cats, pigs and chickens [49, 50, 151]. Small fish like bleak, gudgeon, minnow, smelt, stone loach and sunbleak have traditionally been caught for use as bait to attract large predatory fish in many parts of Europe and nowadays are mainly caught by recreational fishermen for use as live bait. This is a topic for further research [36, 39, 46, 49, 50, 55, 143].

An unusual utilization of a fatty fish is to use it as a candle or torch by inserting a wick and lighting it. The fisher community at Vinön in Lake Hjälmaren in Sweden used dried smelt for that purpose [150]. Fish oil could be extracted by boiling fish, for instance three-spined sticklebacks in the Stockholm Archipelago in the eighteenth century. Besides using the oil for medicine and grease, it was used for simple lamps [50].

These examples reflect the rich variety of how rural communities in Europe have utilized fish as raw materials other than food. There is much scope for further research.

#### Medical uses

Some fish were thought to have healing properties. The tench was often called ‘the doctor fish’; the thick mucus covering the body was thought to heal wounds, a belief which may have been founded on seeing other fish rub themselves along its sides. Tench were often put into store ponds to provide a medical service for other fish [50]. Fish have a pair of small bones in the skull, known as otoliths (composed of aragonite and used for balance and hearing), which were also used in medical preparations. Barbel roe was another country remedy, taken as an emetic and cathartic, as recorded in the nineteenth century [152].

In traditional medicine in Spain, the eel has a historic connection as an ingredient; its blood, bile, fat and meat was used to treat alcoholism, obesity, mental disorder, gout and childbirth. Carp bile, bones and swim bladders are used for treating skin rashes and lumbago, bones and the swim bladder of the Andalusian barbel is also used to ease lumbago. Perch vertebrae are worn as a protection against malaria, parts of the head are used to treat carcinomas and help expel afterbirth while the otoliths are part of a purge for kidney stones. A brown trout tail held in an infant’s mouth promotes language skills. The wels catfish has variety of uses; its head, flesh and liver are treatments for a range of disorders of the skin, intestines and throat [153]. It is interesting that carp (introduced in the seventeenth century to Spain) and particularly wels and perch, both introductions of the 1970s, all play an important role in fish-based ethnomedicine despite their relatively recent introduction to Iberia [154].

The bitterling, a small cyprinid, was used in pregnancy testing; the fish was injected with urine; if the woman was pregnant, the ovipositor of the fish would protrude. Native to central and eastern Europe, it was introduced to Britain in the 1920s, and in the 1930s many fish were collected in the Liverpool area for this medical use [147].

### Confining fish

#### Keeping fish in wells

An interesting practice known in Finland and Sweden and probably also elsewhere in Europe is to keep fish in wells. The fish kept the drinking water clean from frogs and bugs and in Sweden there are reports of keeping pike, crucian carp and eel in this manner [155]. A more detailed study has been carried out in Finland where the so-called *kaivohauki* ‘well pike’ referred to a fish, in most cases a pike, kept in wells or springs from which the drinking water for the house was drawn. Keeping a well pike has been a real tradition in many places in Southwest Finland (Varsinais-Suomi); the pike would eat the woodlice and other insects in the well and keep the water clean. If the pike died or the water was possibly contaminated or even poisoned, then the well was drained [156]. This custom has probably existed elsewhere in Europe, but is very little documented.

#### Fishponds

One way of keeping fish fresh is to store them live in ponds. Much has been written on keeping fish in a live store for the table, which developed into a sophisticated system of ‘pond culture’ [9, 155, 157]. All sorts of enclosed bodies of water, namely ponds, moats and lakes, have been used in historic times where fish were stored and fattened on kitchen and brewery waste. Cyprinids were ubiquitous, with the common carp introduced westwards from its Danubian native range and well established in France by the mid thirteenth century. Carp replaced bream as the fish of choice; pike, perch and eels were also commonly kept. On private country estates belonging to royalty, aristocrats and the wealthy, fish such as carp were grown on to a large size and used in wealth displays and gift exchange. Monasteries needed fish supplies for the many fast days and monks became expert in pond culture, but freshwater fish were mostly for the Abbot’s top table or feasts. Secular estates in the eighteenth century used their ponds as amusement for guests to angle, while the netting of fish was a spectacle for guests with the fish later served at table. At a more modest level, rural dwellers continued to fish river and ponds both commercially and for subsistence [158].

**Fig. 14:**
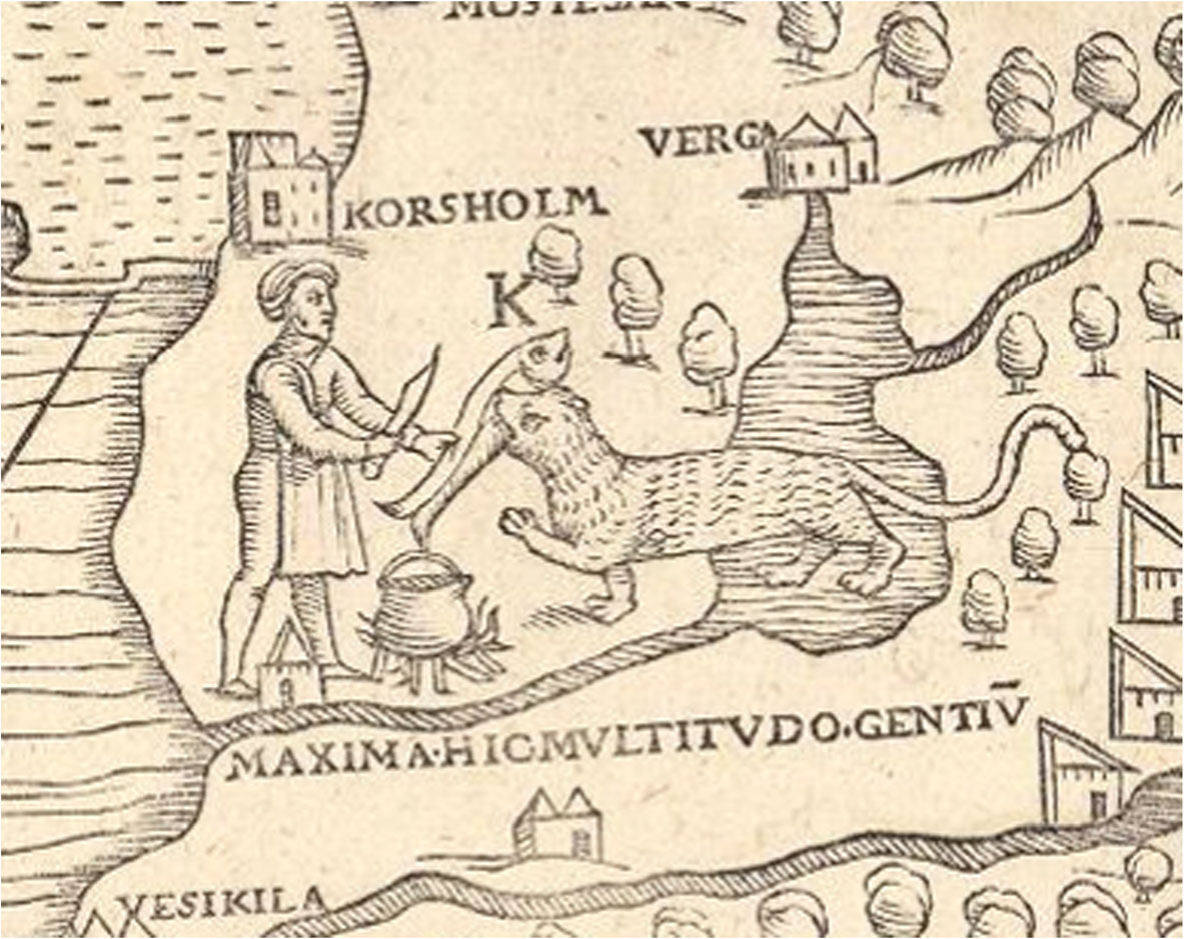
Otter assisting his master in fishing (Olaus Magnus Carta Marina, 1539)

Further north, in Scandinavia, the crucian carp, smaller, but more tolerant of cold temperatures than common carp, was the most popular fish. A less likely species, the stone loach was kept in royal Swedish fishponds in the seventeenth century [155, 159]. Many ponds in urban settings and in vicarages in northern Europe were used as store ponds (Fig. 14).

Although literature on pond culture, such as the work of Peder Månsson in Vadstena Abbey, Sweden in the 1520s [160], Jan Dubravius 1599 [160, 161] in Bohemia and Roger North in England 1714 [162], describe the grading of fish by size, we have no (as yet) European equivalent of a Neolithic assemblage of common carp that was found in China. Here, the ancestral home of early pond culture, the metrical analysis of a large sample of over 500 carp pharyngeal bones revealed different size/age classes suggesting a managed pond system [163], whereas European samples to-date are too small and not contextually linkable to particular pond systems. The Swiss assemblages referred to earlier do indicate some selective lake fishing by size [8], but have not been linked to pond culture. The largest archaeological samples of carp and other freshwater fish tend to come from sites in Eastern Europe along the Danube where from prehistoric times large fish were caught in the river, but were not managed stock [164, 165].

From medieval store ponds, a more sophisticated European aquaculture developed and the Eastern European focus on carp is still evident. The 2012 FAO figures show of the six major finfish species which are headed by salmon (72%) and rainbow trout (12%) carp are fifth (3%); the remaining species are marine. The countries leading carp production are Poland, the Czech Republic and Hungary; in the Czech Republic, the Trebon ponds, in an area formerly described as Southern Bohemia, have a long history and carp are still 90% of the catch. There is evidence that tastes are changing away from buying the whole, sometimes live, fish to prepare at home. The European preference for the convenience of fillets, and other species is beginning to prevail even in areas in Eastern Europe where carp was a traditional Christmas dish and the purchase of whole, even live, freshwater fish remained popular longer than in the west [166].

Brown trout do not appear to have been commonly considered prime pond fish in the later medieval period in Western Europe, though they were kept in Poland [167], their need for high oxygen levels and flowing water made them unsuitable for small ponds. In Western Europe, trout ‘farming’ did not really develop until the mid nineteenth century following the work of two French fishermen, Géhin and Remy from La Bresse, whose work on artificial fertilisation was published in 1851 and intended for restocking trout rivers rather than pond culture. This stimulated a period of intense interest in many countries in the artificial breeding of fish by egg stripping and the French government built the Huningue facility near Basel for this purpose. In fact, the same methods had been published a century before by Ludwig Jacobi in the *Hanover Magasine* (1763), while an Italian monk had been carrying out similar experiments, but these seem to have been forgotten. Better suited to pond culture was the rainbow trout introduced from North America in 1882, initially for recreational purposes but soon became an important fish in the food supply [167].

Ornamental fish were to become a European fashion, the earliest being the goldfish, first brought from China most likely via Portugal (from the Portuguese trading post in Macau) in the seventeenth century. The actual date of their European debut is disputed, but this fish had a long history in China where different varieties had long been bred and displayed in ponds and porcelain jars. In Europe, it was initially a fish of indoor aquaria in public spaces and private wealthy homes; however, the hardy standard form was soon to be found not only in the prison of a glass bowl but also in outdoor ponds where in suitable temperatures it could breed. Other prized ornamental fish included golden forms of the orfe (also known as the ide) and tench, both cyprinids. Koi carp were a much later introduction to Europe, most likely developed in Japan not much earlier than the 1800s, though coloured carp had been known for much longer in China and Japan. There was some interbreeding with German mirror carp and today thirteen colour forms are known with fish judged on a number of characteristics including colour, form, size and perfection of scales. They were introduced into Europe in the 1950s and became more popular as airfreight became a viable way of transporting the fish to a growing market [9, 50, 168].

In the mid nineteenth century, Pierre Carbonnier founded a public aquarium in Paris and was the first to breed a tropical species in Europe; the Paradise fish from stock brought from southeast China in 1869. In the 1870s, he imported the first Siamese fighting fish and other species well known to aquarists today. Thanks to technical progress during the nineteenth century, public aquariums were established at about the same time as the zoological gardens in Europe. Public aquariums opened up in London 1853, Paris 1860, Vienna 1860, Hamburg 1864, Berlin 1869, Napoli 1874 and Stockholm 1891 [169, 170].

In the early European aquarium trade in the late nineteenth century, small native freshwater species from Central and South Europe were also in demand. These included the European mudminnow and the weatherfish (said to react to stormy weather conditions); they were caught in large numbers in Hungary [87, 102]. There is evidence of keeping weatherfish in ponds in the eighteenth century [171]. Native European fish species for pond or tank keeping are still to be found in the aquarium trade [172].

#### Introduction of fish (biotic globalisation)

As well as ornamental fish, many other species have been introduced into new countries and new continents for food and sport, for example the common carp, and the brown trout to the Americas and Australia and New Zealand, often under the auspices of the Acclimatisation Societies, that became popular in the nineteenth century. The mosquito fish [173] was introduced into Spain in the early twentieth century and then to Italy in 1922, as a form of mosquito control and then all over southern Europe. It reached Corsica in 1924, Istria in 1925, Dalmatia in 1926 and Greece in 1928. By the early 1930s, it had been introduced almost over all southern Europe up to Romania and Hungary. The mosquito fish was also introduced into the Soviet Union (Daghestan, Caucasus) in 1924 [174].

Other introductions were earlier, in England for example the carp and the goldfish as shown above, which soon embraced English waters outside captivity. Native fish have been introduced into local waters where they were previously not established, often by anglers as bait (such as the minnow) or to stock angling lakes as recreation fishing becomes increasingly lucrative. Fish have independently expanded their range via canals; the great age of canal building began in the eighteenth century.

Today, the introduction of new species is often regarded as harmful, and some species are categorized as invasive [175]. More recently, escapes from fish farms, angling lakes, dumping of unwanted pets and accidental transport in ship ballast have caused conflict with the native fauna, upsetting the food chain or carrying diseases native fauna cannot combat. The topmouth gudgeon, originally from Asia and introduced in to the UK in the 1980s for aquaria, soon left these confines and is now found in lakes where it parasitizes other fish eating their scales and flesh. It is now illegal in the UK, but difficult to eradicate and has also been a successful invasive alien elsewhere in Europe [176, 177]. A larger fish but a smaller problem in England is wels or the Silurian catfish; a Danubian native, it is now found across western Europe and grows very large, well over 2 m, under the right conditions. Eaten in the east but more of a specialist anglers’ fish westwards, its voracious and indiscriminate appetite can be problematic for other fauna. Introduced in England in the mid nineteenth century, it has never become a major pest nor grows very large (though warming temperatures may change this). It has been contained by restriction to closed waters under licence. Any found in open waterways must be reported, captured and relocated [9, 178].

A recent study on the High Pyrenees mountain lakes showed the detrimental effect of introduced brown trout and the minnow (a small cyprinid) on amphibians and zooplankton in these formerly pristine waters [175]. While the brown trout can be detrimental in new waters, this fish can be adversely affected by the introduction of other trout. They have very diverse genetic forms, which can be visually separated by colour patterns and size as well as genetically. Often these forms are specific to a region or water body. The introduction of trout from elsewhere to raise stocking levels can be deleterious to genetic integrity through interbreeding. Recent taxonomic studies in Italy [179] on trout of different ‘types’ in different habitats—defined as Mediterranean trout (Alps, Apennines and main islands), marbled trout (Po Valley) and the large ‘carpione’ trout (Lake Garda), have highlighted the problems of restocking by mixing these populations and importing ‘Atlantic’ trout from farther west weakening their unique genetic codes. Farmed trout in particular are a problem and most fisheries biologists now recommend farmed stock should be sterile. These fish are not just genetically different to their wild counterparts but also behaviourally quicker to become sexually mature and easier for the angler than their wild cousins.

#### Recreational fishing

Subsistence fishing in lakes, rivers and streams is rare nowadays in many places of Europe, and has in general been replaced by recreational fishing. Artificially stocked lakes, with cyprinids (of which the common carp is king) and also trout are successful commercial ventures, especially if specimen fish are promised. Even the wels catfish has its own devotees who seek the largest specimens, the River Ebro in Spain is a favoured location. Clean and unspoilt stretches of rivers with salmon and trout command high prices as fly fishermen seek a rural idyll. The ultimate is wild fishing where a guide takes the angler seeking something a little more challenging to remote waters in raw nature. All these have become part of a growing income source in regions where other traditional income streams have diminished. In many ways, recreational anglers have become the guardians of freshwater fish stocks with investment, the practice of catch and release, maintaining waters and the surrounding landscapes. However, the introduction of fish stock and the management of the fishing experience becomes an increasingly cultural construct geared to guarantee satisfaction. Apart from ‘wild fishing;, it takes places in ‘managed nature’ and the constant movement of fish to stock waters has genetic implications as described above [9].

#### Harvesting other aquatic organisms

In the 1760s, medicinal leeches became widely accepted as a medicament and demand increased all over Europe. By the 1830s, around 50 million leeches were employed in hospitals every year causing a shortage of leeches. For instance, in 1830, the yearly average of leeches used amounted to 5 to 6 million at the Hôtel Dieu in Paris, and 7 million in London. The annual usage in Russia reached about 30 million. Leeches were harvested in many locations in Europe. In the Hungarian marshes, the Pákász, who were specialised in capturing aquatic organisms, gathered leeches for the European trade. They were sold to France, Sweden and elsewhere [87]. In the nineteenth century, this demand resulted in over-exploitation and reduced many local populations. Breeding medicinal leeches in ponds became a going concern in many countries. Several farms were founded in rural and urban settings in France, Germany, Denmark and Sweden. Our knowledge about this culture is rather scant, but toponyms serve to remind us of such ponds [180].

Freshwater bivalves provide an ecosystem service as filters in lakes and rivers, but they have also been used as human food and for making simple tools in some parts of Europe. Archaeozoological remains show that freshwater bivalves were eaten in Central and Northern Europe during Neolithic times [181] and much more recently. The swan mussel was still gathered and eaten by peasants on the Baltic Island of Gotland in the nineteenth century and also in the province of Småland. In the 1840s, freshwater bivalves were used as famine food in Ireland [182]. There are also records from the Polish town Brżezany (contemporary Berezhany in Ukraine) that poor people gathered large amounts of bivalves and cooked them together with cereals as food. It seems to have been common in Italy to gather freshwater bivalves as food locally, while in Ottweiler near the city of Trier (Germany), in the early twentieth century children still gathered large amounts of the thick-shelled river mussel (now endangered) and other mussel species as food. In the early years after World War II, a type of sausage, the Berliner Muschelwurst (made from mussels), was bought by the inhabitants of Berlin [183]. The use of freshwater bivalves as human food is worth further exploration. Bivalves were also used as swine and chicken fodder in Central Europe [183]. The shells could also be used as tools or burnt for calcium. Their use as tools is known from nineteenth century Sweden and Germany [182].

While the use of bivalves as food, animal feed and for making tools is still very little explored, fishing for fresh-water pearl mussels is better known. It is nowadays an endangered and regionally extinct species in European waters. It has been widely gathered for its pearls and has been important for local economies in many parts of Europe. During the Middle Ages in Europe, these were the source of the decorative pearls used by nobility on their garments and by the Church for decorating sacred vessels and reliquaries. ‘Pearling’ and its culture has been studied by biologists and historians in northern Fennoscandia, northern Russia, Vogtland, Scotland and elsewhere [35, 184, 185]. Illegal harvesting still occurs in some parts of Europe.

**Fig. 15:**
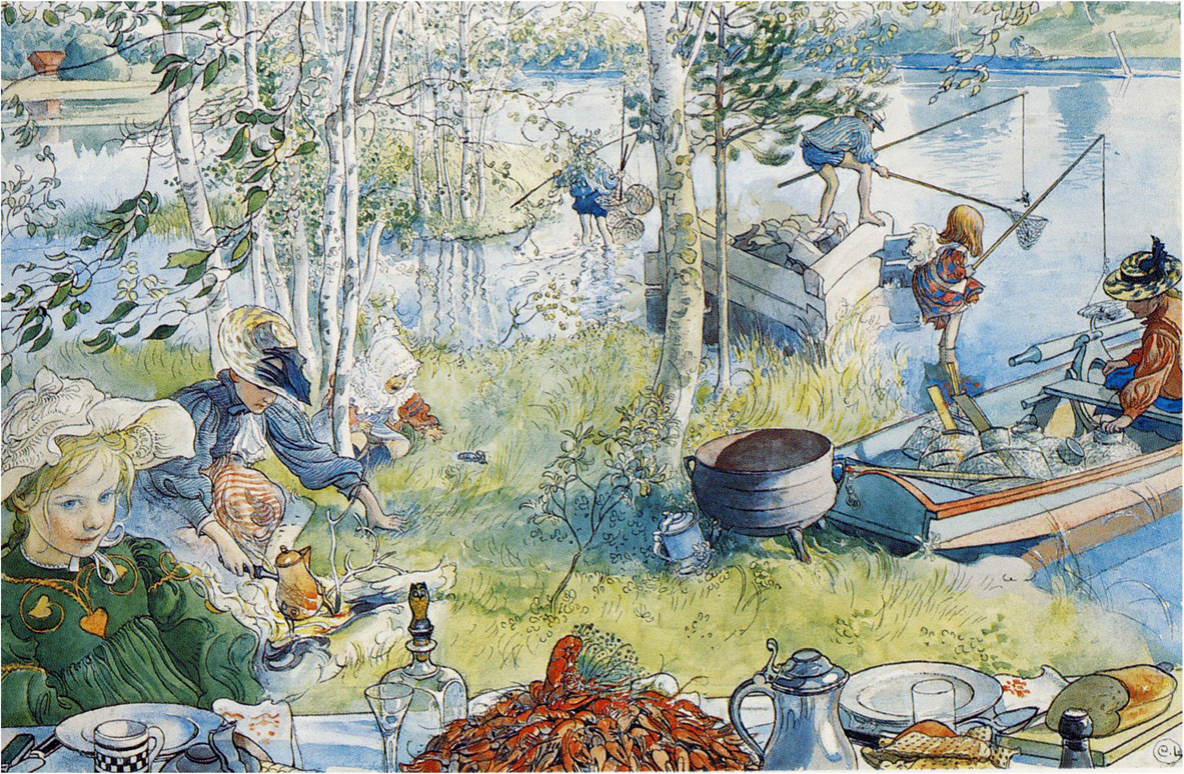
Fishing for crayfish in the 1890s. Painting by Carl Larsson (From C. Larsson, Ett hem, 1897)

There are a number of crayfish species native to Europe including the European or Noble Crayfish. They have been used as human food but also as animal fodder. In parts of Sweden, Finland and Norway, it is common to have crayfish parties with friends. Nowadays, the native European crayfish is almost extinct due to crayfish plague, which almost entirely wiped out the species in many European waters. It has been replaced by alien species or substituted in the trade by imported crayfish from other countries adding extra pressure on native species [186] (Fig. 15).

The tradition of crayfish parties in Scandinavia started in the eighteenth century as an upper-class custom but has since spread more widely. However, fishing for crayfish has been the preserve of artisanal fishermen in rural areas and strictly regulated. A specific culture about gear, baits and way of fishing developed, which has been studied by ethnologists [187], as has the development of crayfish parties [186]. The history and use of crayfish has also been researched in Central Europe [188]. In Britain, the native white-clawed crayfish is threatened by the American signal crayfish introduced in the 1970s to export to the Scandinavian food market. The latter soon escaped confinement and colonised rivers out competing the native species being larger and more voracious. They also carry a fungal disease fatal to the white-clawed crayfish [189].

There are of course other aquatic products and organisms that have been harvested by the European peasantries, including algae, common reed, club-rush and water plants as fodder, or for house roofs and other uses. Seaweed was harvested as manure in the bays and islands of the Baltic Sea and coastal fisherfolk developed interesting taxonomies of their usefulness. However, this is beyond the remit of this article [36, 45, 190].

## Discussion

The ethnobiologist is interested in the dynamic interactions between people, biota and the environment across time and all over the world; this is a common subject in ethnobiology [191, 192]. One important aspect is the relationship between humans and freshwater fish in Europe. The selective data we have presented provides a broad but by no means exhaustive review of traditional European fisheries from the later medieval/early modern period. Earlier medieval documentary data on European water systems and fisheries have been extensively researched by the historian Richard Hoffmann [193] and revealed evidence of the negative effects of pollution, over-fishing and water management on fish stocks. The bone evidence from Flanders referred to above is supportive evidence for the degradation of freshwaters as human population density increased [91] and remains an on-going challenge.

We have highlighted a vanishing world as rural communities are increasingly interconnected to global food supplies, eroding their fishing and culinary history. In the past, even a relatively insignificant fish like the ruffe has made a cultural and culinary contribution [142]. Many traditional fisheries, if they survive, become living history, a tourist attraction, providing extra income for fishermen and communities marginalised by modernity and overfishing. Despite globalisation, there is an increasingly narrow demand for fish in Europe—primarily cod, tuna and salmon, the latter largely farmed, while cod and tuna stocks remain precarious.

As inland freshwater fisheries have declined angling has boomed, both coarse and fly. Leisure has proved more profitable than supplying food from freshwater fisheries. Introducing fish to new waters has globalised distributions with some species particularly adaptable such as the common carp and Nile tilapia, known in aquaculture as the chicken of fish as it fattens quickly and can be ‘farmed’ in large numbers. Bar some catastrophic event, it is difficult to imagine a reversal where artisan fishing in Europe would supply a significantly bigger share of the market than at present. However, as has been shown in domestic animals in regard to the genetic integrity of rare breeds, which are resilient in situations where animals overbred for meat or milk yield would struggle, we need to value these ‘relicts’ of the past. Small-scale artisan fisheries also have much to contribute with regard to community-based management where local control ensures that fisheries can adapt so fish stocks remain viable through good and bad years. Personal investment in safeguarding a resource is often much more successful than government regulation alone. The recording by ethnographers of knowledge passed on and still practiced by generations of fishers are more than a data store, or living history, they are a valuable source of data to inform current fishery policies [50].

## Conclusion

Fish and other aquatic resources have always been utilized everywhere by man. Nonetheless, most ethnobiologists working with European issues are still focusing their research interests on the use of plants. However, in order to develop ethnobiology as a useful discipline with its own identity, we have to widen our interest to include other organisms, especially animals. Fish were important for human nutrition in the past and today. This paper demonstrates the range of research already achieved and highlights areas for future study that will bring ethnoichthyology into the mainstream of ethnobiology complementing European ethnobotanical studies and other forms of data.

## Data Availability

All data generated or analysed during this study are included in this published article.
